# Parallel Improvement of Both Mental and Cardiometabolic Health in Children and Adolescents with Overweight and Obesity, Following the Implementation of a Multidisciplinary Lifestyle Intervention Program

**DOI:** 10.3390/nu18010150

**Published:** 2026-01-01

**Authors:** Aikaterini Vourdoumpa, George Paltoglou, Maria Manou, Diamanto Koutaki, Penio Kassari, Marina Papadopoulou, Gerasimos Kolaitis, Evangelia Charmandari

**Affiliations:** 1 Division of Endocrinology, Metabolism and Diabetes, First Department of Pediatrics, National and Kapodistrian University of Athens Medical School, ‘Aghia Sophia’ Children’s Hospital, 11527 Athens, Greece; katvourdouba@gmail.com (A.V.); mariamanou93@hotmail.com (M.M.); mk_madw@hotmail.com (D.K.); peniokassari@gmail.com (P.K.); marinageorpap@gmail.com (M.P.); 2 Division of Endocrinology, Diabetes and Metabolism, Second Department of Pediatrics, National and Kapodistrian University of Athens, “P. & A. Kyriakou” Children’s Hospital, 11527 Athens, Greece; gpaltoglou@gmail.com; 3 Center of Clinical, Experimental Surgery and Translational Research, Biomedical Research Foundation of the Academy of Athens, 11527 Athens, Greece; 4 Department of Child Psychiatry, National and Kapodistrian University of Athens Medical School, ‘Aghia Sophia’ Children’s Hospital, 11527 Athens, Greece; gkolaitis@med.uoa.gr

**Keywords:** youth, overweight, obesity, lifestyle intervention, cardiometabolic health, CBCL, YSR, mental health

## Abstract

**Background**: Overweight and obesity in childhood and adolescence represent one of the most significant public health challenges of our century. Affected children and adolescents often face psychosocial maladaptation, including low self-esteem, depressive and anxiety symptoms, and behavioral problems, many of which may persist till later in life. The aim of our study was to evaluate the impact of a multidisciplinary, personalized lifestyle intervention program on psychosocial and behavioral symptoms, assessed through standardized psychometric questionnaires, and to investigate their relation with cardiometabolic parameters in children and adolescents with overweight and obesity. **Methods**: In this prospective cohort study, 537 children and adolescents (6–18 years; females: 52.9%; pubertal: 43.6%) with obesity (n = 44.3%), overweight (n = 33.7%), or normal BMI (n = 22%) participated in a personalized lifestyle intervention program for one year. Clinical and laboratory evaluations, including anthropometric, cardiometabolic, and endocrinologic parameters, as well as psychosocial functioning assessed by the Child Behavior Checklist (CBCL) and Youth Self-Report (YSR), were performed at the beginning and the end of the study. Linear regression analyses identified predictors of psychometric change. **Results**: At initial evaluation, children and adolescents with obesity displayed a less favorable cardiometabolic profile and greater emotional/conduct difficulties compared to their overweight and normal-BMI counterparts. Following the intervention, significant improvements were observed in BMI, anthropometric and cardiometabolic parameters, as well as reductions in internalizing, externalizing, and total problem scores across multiple CBCL and YSR domains (*p* < 0.05). The improvements in psychosocial functioning were partly independent of BMI reduction. Linear regression analyses identified cardiometabolic and endocrine markers as significant predictors of psychometric change (*p* < 0.05), highlighting interactions between metabolic recovery, pubertal hormones, and stress physiology. **Conclusions**: A personalized, multidisciplinary lifestyle intervention program implemented for 1 year led to parallel improvements in psychosocial and cardiometabolic health in children and adolescents with overweight and obesity. Identification of specific metabolic and endocrine predictors provides novel insights into potential biological mechanisms associated with adiposity, emotional well-being, and neurodevelopment.

## 1. Introduction

Childhood obesity has emerged as one of the most significant public health challenges, reaching epidemic proportions and affecting approximately 20% of children and adolescents worldwide [1]. The prevalence of overweight and obesity in Greece is 21% in preschool years and 41% in children aged 6–18 years, and is significantly higher than the prevalence of overweight and obesity in other European countries (15% and 25%, respectively) [2]. Childhood obesity is a complex, chronic condition that arises from the interaction of genetic predisposition, epigenetic modifications, and environmental influences, such as diet, physical inactivity, and sleep disturbances, with genetic susceptibility exerting its strongest impact during early life [3,4].

Multiple comorbidities emerge, and beyond its well-established associations with insulin resistance, dyslipidemia, hypertension, and other cardiometabolic complications [5,6], childhood obesity is increasingly recognized as a multidimensional disorder involving neuroendocrine dysregulation, and psychological and behavioral maladjustment [7,8,9]. Visceral fat accumulation resulting from excess energy intake results in a state of chronic low-grade inflammation through increased secretion of proinflammatory cytokines, such as Tumor Necrosis Factor-α (TNF-α) and Interleukin-6 (IL-6), and altered adipokine secretion, including increased pro-oxidative leptin concentrations and counter-regulatory anti-oxidative adiponectin concentrations [10,11]. This “pro-inflammatory” state contributes to insulin resistance, dyslipidemia, hypertension, gut dysbiosis, hypothalamic–pituitary–adrenal (HPA) axis dysregulation, and endothelial dysfunction, thereby accelerating cardiometabolic risk from early life [11]. More importantly, systemic inflammation and metabolic imbalance also affect the central nervous system [9]. Inflammatory mediators and altered hormonal signaling may disrupt hypothalamic appetite regulation, impair dopaminergic and serotonergic pathways, and promote neuroinflammation in regions critical for emotion and cognitive control, such as the prefrontal cortex, amygdala, and hippocampus [12,13,14]. These mechanisms associate obesity not only with metabolic complications but also with an increased risk of depression, anxiety, and emotional/conduct difficulties in children and adolescents [9,11,12,13,14,15].

Notably, epidemiologic data indicate that body mass index (BMI) during childhood correlates positively and independently with the risk of coronary heart disease in adulthood—specifically between ages 7–13 years for boys and 10–13 years for girls—and that normalizing BMI before the onset of puberty attenuates this increased risk significantly [7,16]. Furthermore, children and adolescents with excess adiposity exhibit higher rates of internalizing symptoms (e.g., depression, anxiety, somatic complaints), externalizing behaviors (e.g., aggression, rule-breaking, attention problems), and reduced social competence compared to peers with normal BMI [8,17,18,19]. Increasing evidence suggests that disruptions in stress physiology contribute to this association [20,21,22]. Children with obesity often display dysregulation of the HPA axis, characterized by heightened stress reactivity and interindividual variation of cortisol concentrations (mildly elevated, normal, or even low basal cortisol concentrations) [20,21]. These alterations enhance visceral fat deposition, promote low-grade inflammation, and affect neural pathways involved in emotional regulation, reward processing, and impulse control. Consequently, stress-related biological changes may predispose children to emotional dysregulation, stress-eating behaviors, and greater vulnerability to internalizing and externalizing symptoms [20,21].

The bidirectional relation between childhood obesity and mental health is increasingly emphasized. Emotional dysregulation, impulsivity, and maladaptive coping behaviors contribute to overeating, sedentary lifestyle, and weight gain, while obesity itself worsens psychosocial outcomes through stigma, body image disturbances, and alterations in neuroinflammatory pathways impacting mood regulation [23,24,25,26,27]. Lifestyle interventions combining dietary modification, structured physical activity, behavioral counseling, and family education are considered the first-line management for childhood obesity [28], with digital technologies offering additional support to optimize adherence and outcomes [29,30]. Interestingly, the MEND 7–13 study included 3782 children participating in a 10-week, twice-weekly community-based program, and demonstrated significant short-term improvements in BMI, BMI z-score, cardiovascular fitness, and psychological outcomes [31]. Similarly, in the Bright Bodies randomized controlled trial, 209 children with overweight were enrolled in a 12-month family-based lifestyle program, resulting in sustained reductions in BMI, body fat, and insulin resistance relative to standard care [32].

However, despite evidence that lifestyle interventions can improve metabolic parameters [33] and reduce inflammatory markers [34,35], previous studies have rarely incorporated long-term, personalized, multidisciplinary programs, nor have they systematically evaluated psychosocial functioning alongside metabolic and endocrine markers to identify biological predictors of psychological change [36,37,38]. Based on the above, the aim of this study was to evaluate the effects of the implementation of a multidisciplinary, personalized, lifestyle intervention program for one-year on psychosocial and behavior difficulties, and to investigate their relation with cardiometabolic and endocrine parameters in children and adolescents with overweight and obesity.

## 2. Patients and Methods

### 2.1. Study Design and Patients

Five hundred and thirty-seven (n = 537) children and adolescents aged 6–18 years (mean age ± SE: 10.15 ± 0.11 years; females: 52.9%; prepubertal: 56.4%) were prospectively recruited to participate in our study. The participants were consecutive attendees at the Center for the Prevention and Management of Overweight and Obesity in Childhood and Adolescence, Division of Endocrinology, Metabolism, and Diabetes, First Department of Pediatrics, National and Kapodistrian University of Athens Medical School, ‘Aghia Sophia’ Children’s Hospital. Subjects were classified as having obesity (n = 238, 44.3%), overweight (n = 181, 33.7%) or normal BMI (n = 118, 22%) according to the International Obesity Task Force (IOTF) criteria [39,40,41], and were enrolled in a structured, multidisciplinary, personalize, lifestyle intervention program for at least one year, with data collected both at initial and at the annual follow-up assessments.

Exclusion criteria included age below 6 years, the presence of a diagnosed neuropsychiatric or developmental disorder, and known monogenic forms of obesity. The study complied with the principles outlined in the Declaration of Helsinki and received ethical approval from the Committee on the Ethics of Human Research at ‘Aghia Sophia’ Children’s Hospital (approval number: EB-PASCHMoM: 28 November 2013, ref: 10290-14/05/2013 and approval number: EB-PASCH-MoM: 3 April 2018, ref: 7000-20/03/2018). Written informed consent was obtained from parents or legal guardians, and assent was given by children older than 7 years. Children and adolescents who did not enter the study were treated with the same standards of healthcare.

### 2.2. Intervention Protocol

All participants were admitted to the Endocrine Unit early in the morning of their initial visit. Upon admission, a comprehensive medical history was taken, followed by a detailed clinical examination. Standard anthropometric measurements were obtained by a single trained observer, and baseline blood samples were collected at 8:00 a.m. after a 12 hour (h) overnight fast for hematologic, biochemical, and endocrinologic investigations. In addition, two psychometric questionnaires were completed, the Child Behavior Checklist (CBCL) and the Youth Self Report (YSR) for subjects 6–18 years old and 11–18 years old, respectively. Participants were then enrolled in a 1-year multidisciplinary, lifestyle intervention program designed to address overweight and obesity. This program offered personalized recommendations and guidance on healthy diet, quality of sleep, and regular exercise, tailored to both the participants and their families, as described in previous studies [19,42,43,44,45,46].

The intervention program involved regular evaluations by a pediatrician, pediatric endocrinologist, pediatric dietitian, professional fitness trainer, and, when necessary, a pediatric psychologist. The lifestyle program’s protocol included monthly follow-up evaluations for participants with obesity, bi-monthly evaluations for those with overweight, and quarterly follow-up evaluations for those with normal BMI. Each follow-up visit included a multidisciplinary evaluation with determination of anthropometric parameters, clinical assessments, a 24 h dietary recall, and a review of previously established goals. At the end of the 12-month program, participants underwent again detailed hematologic, biochemical, and endocrinologic investigations at 8:00 a.m. following a 12 h overnight fast and completed the two psychometric questionnaires, CBCL and YSR.

Specifically, the pediatric dietitian assessed the daily eating habits of participants and conducted a 24 h dietary recall using the USDA method [47]. Based on this information, a personalized dietary plan was developed, taking into account individual food preferences, accessibility, whether meals were prepared at home or purchased, and daily routines. Participants were encouraged to follow a balanced meal plan comprising of three main meals (breakfast, lunch, and dinner) and two healthy snacks (such as fruits or vegetables) per day. This plan adhered to the “My Plate” model from the 2010 USDA guidelines and the National Nutritional Guide for Infants, Children, and Adolescents [48,49]. The fitness trainer documented participants’ hobbies and activity preferences, collaborating with families to create enjoyable and tailored physical activity plans. Families were also encouraged to minimize sedentary behavior and engage in daily physical activities for 30–45 min, such as walking, cycling, or dancing. Additionally, a pediatric psychologist provided support to participants and their families when needed.

Sleep recommendations were customized according to age, based on the American Academy of Sleep Medicine Consensus Guidelines [50], suggesting 9–12 h of sleep for children aged 10–12 years and 8–10 h for adolescents aged 13–18 years. Participants were advised to prioritize uninterrupted sleep, aim for an early bedtime (preferably before midnight), and maintain a consistent sleep schedule. Furthermore, limitation of screen time to under two hours per day and power down electronic devices at least one hour before bedtime was encouraged.

Among the 537 recruited children, follow-up data were available for 406 participants (25% dropout rate), primarily due to loss to follow-up, limited adherence to scheduled visits or withdrawal from the intervention for personal or family reasons. Program compliance was monitored through attendance at follow-up appointments. All participants completing the study attended scheduled multidisciplinary evaluations and received continuous guidance on diet, physical activity, and sleep, thereby demonstrating high adherence to the protocol.

### 2.3. Anthropometric and Body Composition Parameters

Body weight was measured with participants dressed in light clothing and without shoes, using a standardized scale (Seca GmbH & Co. KG, Hamburg, Germany). Standing height was recorded in a similar manner, without shoes, using a Harpenden stadiometer (Holtain Limited, Crymych-Dyfed, UK). BMI was calculated as weight in kilograms (kg) divided by height in meters squared (m^2^), with BMI z-scores derived based on Greek standard growth charts [51]. Waist and hip circumferences were assessed according to the WHO STEPwise approach to surveillance (STEPS) protocol, employing a stretch-resistant measuring tape (Seca GmbH & Co. KG, Hamburg, Germany) while participants stood upright [52]. Systolic (SBP) and diastolic (DBP) blood pressure measurements were obtained twice using a sphygmomanometer with appropriate age-specific cuff (Comfort 20/40, Visomat, Parapharm, Metamorphosi, Attiki, Greece), and the mean values were calculated. Furthermore, body composition was evaluated using bioelectrical impedance analysis (BIA) with the TANITA MC-780U Multi-Frequency Segmental Body Composition Analyzer (Amsterdam, The Netherlands). This streamlined approach ensured consistency and precision in the collected anthropometric and physiological data.

### 2.4. Assays

Hematologic analyses were performed using the ADVIA 2110i analyzer by Roche Diagnostics GmbH, Mannheim, Germany. Glucose, total cholesterol, triglycerides (TG) and high-density lipoprotein cholesterol (HDL-c) concentrations were measured with the ADVIA 1800 Siemens analyzer (Siemens Healthcare Diagnostics, Tarrytown, NY, USA). Apolipoprotein A1 (ApoA1), ApoB, and lipoprotein (a) [Lp(a)] concentrations were assessed using latex particle-enhanced immunonephelometric assays conducted on the BN ProSpec nephelometer from Dade Behring (Siemens Healthcare Diagnostics, Liederbach, Germany). Hemoglobin A1C (HbA1C) concentrations were determined with an automated glycohemoglobin analyzer HA-8160 (Arkray, Kyoto, Japan) via reversed-phase cation exchange high-performance liquid chromatography.

Thyroid-related parameters, including TSH, FT4, T3, antithyroid peroxidase antibodies, and anti-thyroglobulin antibodies, along with adrenocorticotropin (ACTH), cortisol, insulin-like growth factor-I (IGF-I), and insulin-like growth factor binding protein-3 (IGFBP-3) concentrations, were measured using chemiluminescence immunoassays on the IMMULITE 2000 system (Siemens Healthcare Diagnostics Products Ltd., Surrey, UK). Follicle-stimulating hormone (FSH), luteinizing hormone (LH), estradiol, ferritin, and insulin concentrations were analyzed with an automated electrochemiluminescence immunoassay analyzer (Cobas e411, Roche Diagnostics GmbH, Mannheim, Germany). Total 25-hydroxyvitamin D (25-OH-Vitamin D) concentrations were quantified using an automated electrochemiluminescence immunoassay on the Modular Analytics E170 analyzer (Roche Diagnostics, Basel, Switzerland).

Insulin resistance was calculated using the homeostasis model assessment of insulin resistance (HOMA-IR) method, defined as: HOMA-IR = [fasting glucose (mg/dL) × fasting insulin (mU/L)]/405.

### 2.5. Psychometric Questionnaires

The CBCL and YSR questionnaires are part of the Achenbach System of Empirically Based Assessment (ASEBA), a worldwide acknowledged and widely utilized system in both clinical and research contexts [53]. The ASEBA system has demonstrated validity for application across diverse societies, cultures, languages, genders, and age groups [53,54,55].

Designed by Achenbach and Edelbrock [56], the CBCL is completed by parents to assess their children’s behavior and psychology at the ages of 6–18 years. Conversely, the YSR is completed by the adolescents themselves at the ages of 11–18 years [57]. These instruments can be administered together, as they share a unified structure and thematic framework. Both questionnaires consist of two sections. The first section employs open- and closed-ended questions to ascertain demographic information, activities (sports, hobbies), academic performance, interpersonal relationships, and overall child competencies. The second part incorporates 113 questions in CBCL and 112 questions in YSR, formatted as Likert-type items with three potential responses. Scores range from 0 to 2, indicating increasing levels of emotional intensity (0 = not at all, 1 = sometimes, 2 = very often or frequently). Exclusively in YSR, 16 questions pertain to socially desirable functions. The remaining questions in both tools examine problematic behaviors, forming empirically-based subscales that, in turn, define two broader behavioral domains: Internalizing and Externalizing Behaviors [58]. Furthermore, the CBCL and YSR align with DSM-V diagnostic criteria, with respective subscales formatted accordingly [59].

Numerous studies confirm the reliability and validity of these tools across their hierarchical scales (both empirical and DSM-oriented). These studies demonstrate significant internal consistency, test-retest reliability, and robust content, criterion, and construct validity [60]. The CBCL and YSR were standardized for the Greek population by Roussos et al. in 1999 and 2001, respectively [61,62].

In the present study the YSR questionnaire was completed only by participants older than 11 years.

### 2.6. Statistical Analysis

All analyzed variables adhered to the normal distribution, and the results are expressed as the mean ± standard deviation (SD). A *p*-value of less than 0.05 was considered statistically significant. During the initial evaluation, anthropometric parameters were analyzed across all sample and the separate BMI groups (obesity, overweight, and normal ΒΜΙ), using a one-way analysis of variance (ANOVA). To assess the impact of the lifestyle intervention, all variables measured at the beginning of the study (“initial assessment”) and one year later (“annual assessment”) were evaluated through repeated-measures ANOVA, Chi-square analysis, and Yates correction. Significant main effects were identified using Fischer’s Least Significant Difference (LSD) post hoc analysis. Stepwise forward linear regression models were used to explore predictors of psychometric score change during the intervention. The difference in variables and scores between the two assessments was used in the latter analysis. All statistical analyses were performed using version 4.3.1 of the R Project for Statistical Computing (R Foundation for Statistical Computing, Vienna, Austria).

## 3. Results

A total of 537 children and adolescents (females: 52.9%, males: 47.1%; pubertal: 43.6%, prepubertal: 56.4%) aged 6–18 years (mean age ± SE: 10.15 ± 0.11 years) were recruited prospectively to participate in a lifestyle intervention program for one year. Participants were categorized into three BMI groups: obesity (n = 238, 44.3%), overweight (n = 181, 33.7%) and normal BMI (n = 118, 22%) according to the IOTF criteria (1–3). Table 1 presents the anthropometric, hematologic, biochemical, cardiometabolic risk, and endocrinologic parameters at initial and annual assessments, along with the associations among these variables. Table 2 and Table 3 detail the psychometric scores from CBCL and YSR and the observed correlations between the initial and annual assessment parameters.

### 3.1. Cardiometabolic Risk

Both initial and annual assessments indicated that children and adolescents with obesity demonstrated a less favorable cardiometabolic profile in comparison to their overweight or normal BMI counterparts. Specifically, statistically significant elevations were noted in indicators of cardiometabolic risk and insulin resistance, including BMI, BMI z-score, blood pressure, waist circumference (WC), hip circumference (HC), waist-to-hip ratio (WHR), waist-to-height ratio (WHtR), uric acid, triglycerides, low-density lipoprotein cholesterol (LDL-c), apolipoprotein B (Apo-B), glycated hemoglobin (HbA1C), insulin, and HOMA-IR. In addition, children and adolescents with obesity have significantly lower concentrations of HDL-c, apolipoprotein A1 (Apo-A1), sex hormone-binding globulin (SHBG), and 25-hydroxyvitamin D (25OHD) concentrations, and higher inflammatory markers [erythrocyte sedimentation rate (ESR) and ferritin] compared with their overweight or normal BMI counterparts.

Following the implementation of the lifestyle intervention program for one-year, statistically significant improvements were observed in anthropometric parameters (BMI, BMI z-score, WHtR), cardiometabolic risk factors (LDL-c, HDL-c), and glucose metabolism (HbA1C) in children and adolescents with excess adiposity and the overall sample compared to initial assessment.

### 3.2. Psychometric Questionnaires

#### 3.2.1. Initial Evaluation

At initial assessment, children and adolescents with obesity demonstrated significantly lower scores in social and total competence scales on CBCL compared to peers with overweight or normal BMI. In addition, they had increased scores in multiple CBCL psychometric scales, including Anxious/Depressed, Withdrawn/Depressed, Somatic Complaints, Social Problems, Aggressive Behavior, Internalizing Problems, Total Problems, Affective Problems, Anxiety Problems, Oppositional Defiant Problems, Sluggish Cognitive Tempo, and Post-Traumatic Stress Problems (Table 2). When psychometric scores were analyzed categorically—using established questionnaire thresholds to distinguish between borderline-clinical and normative ranges—participants with obesity continued to display significantly higher scores across several CBCL subscales (Supplemental Table S1), including Withdrawn/Depressed, Social Problems, Externalizing Problems, Total Problems, Anxiety Problems, and Conduct Problems compared with their normal BMI counterparts.

We further analyzed our data according to gender (Supplemental Tables S5 and S6). At initial assessment, males with obesity displayed higher scores across many CBCL scales (withdrawn-depressed, somatic complaints, anxiety problems, sluggish-cognitive problems, post-traumatic stress problems), compared with males with normal BMI. Among females, excess adiposity was associated with elevated scores in aggressive behavior, internalizing and total problem scales compared to females of normal BMI. A comparison between genders revealed that males exhibited higher levels of anxious-depressed, withdrawn-depressed, social, internalizing, affective, anxiety, attention deficit hyperactivity, conduct, obsessive compulsive and post-traumatic stress symptoms compared with female participants.

In contrast, these associations were not observed in the YSR questionnaire (Table 3, Supplemental Tables S2 and S6).

#### 3.2.2. Lifestyle Intervention

The impact of the lifestyle intervention on changes in psychometric scores was analyzed by comparing the initial and annual assessments. Firstly, scores were treated as a quantitative variable, revealing statistically significant improvements in most CBCL subscales (Table 2) among subjects with excess adiposity, as well as in the overall sample, compared to the initial assessment, especially in the internalizing, externalizing, and total problem symptom scales. Similarly, significant improvements were observed in multiple subscales of the YSR questionnaire (Table 3), including anxious-depressed, somatic complaints, social, thought, aggressive behavior, affective, anxiety, and somatic problems and the overall internalizing, and total problem scales. Furthermore, scores were analyzed as a categorical variable by grouping them into two categories: normal score and borderline-clinical score (Supplemental Tables S3 and S4). Notably, following the lifestyle intervention, significant improvements in psychometric scores were also observed across all CBCL subscales and most YSR subscales in children and adolescents with obesity, as well as in the overall sample.

As far as analysis by gender is concerned, following the intervention, males with obesity presented significant improvements in many CBCL and YSR scales (Supplemental Tables S5 and S6), including school, somatic complaints, thought problems, withdrawn-depressed, attention problems, aggressive behavior, externalizing problems, affective problems, somatic problems, attention deficit-hyperactivity problems, oppositional defiant problems, sluggish cognitive problems, obsessive-compulsive problems, and post-traumatic stress problems, compared to initial evaluation. However, comparable psychosocial improvements were not observed in females.

### 3.3. Predictors of Psychometric Score Change

**Figure 1:**
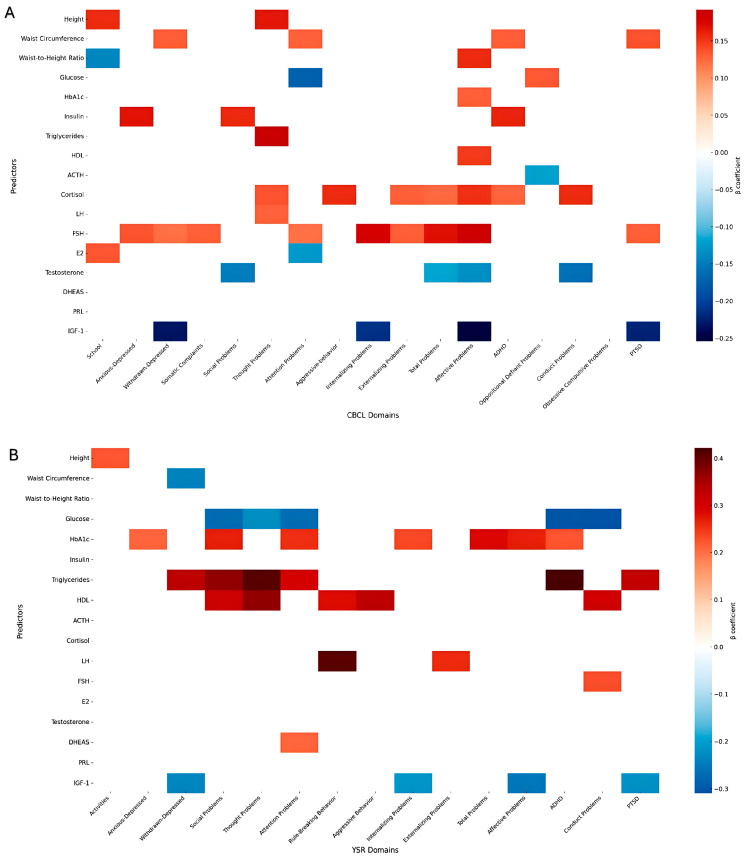
Predictors of psychometric score change during lifestyle intervention based on multiple linear regression models in (**A**) CBCL and (**B**) YSR.

To explore determinants of psychometric score changes during the intervention, stepwise forward linear regression models were applied across five domains: anthropometric, metabolic syndrome, glucose metabolism, pituitary function, and peripheral hormones (Figure 1, Supplemental Tables S7 and S8).

#### 3.3.1. Anthropometric Parameters (Weight, Height, BMI, WC, HC, WHR, WHtR)

Several measures of adiposity predicted changes in psychometric scores. Height was associated with both school performance and thought problems, while waist circumference and waist-to-height ratio predicted a range of outcomes, including withdrawn-depressed, attention problems, affective problems, and post-traumatic stress symptoms.

#### 3.3.2. Metabolic Markers (Glucose, SBP, WC, Triglycerides, HDL-C)

Triglycerides were positive predictors of multiple outcomes, including reduction in thought problems, social difficulties, and attention-related disorders. Change in glucose concentrations had a negative—albeit non-significant—association with the improvement in attentional, social, and conduct problems. Moreover, increase in HDL-c showed positive associations, predicting reduction in affective, thought, rule breaking, aggressive, and social problems.

#### 3.3.3. Glucose Metabolism and Insulin Sensitivity (Glucose, Insulin, HbA1C, HOMA-IR)

Insulin concentrations predicted anxious-depressed symptoms, social difficulties, and hyperactivity, whereas HbA1C was associated with social, attention, anxiety, depressive and affective problems, hyperactivity, and broader internalizing and total problem difficulties.

#### 3.3.4. Pituitary Hormones (TSH, PRL, LH, FSH, ACTH)

Both FSH and TSH were strong predictors, each linked with multiple internalizing and externalizing domains. LH, PRL, and ACTH showed more specific associations, such as thought problems and oppositional defiant symptoms.

#### 3.3.5. Peripheral Hormones (IGF-I, FT4, DHEAS, E2, Testosterone, Cortisol)

Cortisol reduction emerged as a consistent positive predictor of amelioration in thought, conduct problems, externalizing, total problems, hyperactivity, and affective domains, while testosterone was inversely associated with conduct and social problems. DHEAS and IGF-I were associated with affective and internalizing outcomes, while estradiol showed domain-specific associations with school and attention scales.

## 4. Discussion

In the present study, we investigated the effect of a personalized lifestyle intervention program in children and adolescents with overweight and obesity on social competence and problem behaviors, and their association with cardiometabolic parameters. We demonstrated that following the participation in the one-year lifestyle intervention, multiple psychometric scores improved independently of BMI, as well as cardiometabolic risk factors involved in lipid and insulin metabolism. These results suggest that a comprehensive, multidisciplinary, personalized lifestyle intervention program is associated with improvements in the metabolic and psychological dimensions of childhood obesity, independently of the BMI of children and adolescents.

### 4.1. Psychosocial and Cardiometabolic Burden of Pediatric Obesity

Prior to the intervention, children and adolescents with obesity displayed both an unfavorable cardiometabolic profile and significantly greater emotional and behavioral problems on CBCL. To begin with, the identified increases in cardiometabolic risk factors (BMI, BMI z-score, WHtR, LDL-c, HDL-c, HbA1C) reflect a metabolic dysfunction commonly observed in childhood obesity, where increased visceral fat deposition drives insulin resistance, lipid imbalance, and systemic inflammation, thereby contributing to early atherogenic changes and features of metabolic syndrome [38,63,64,65,66]. Moreover, elevated internalizing (e.g., anxious/depressed, withdrawn) and externalizing (e.g., aggressive, oppositional) scores, as well as reduced social and overall competence, were identified. These results are consistent with previous studies demonstrating a strong association between childhood obesity and increased risk for psychosocial maladjustment [17,18,19]. Indeed, obesity in adolescent girls has been associated with subsequent decreased academic achievement, reduced earnings, and lower rates of marriage [67]. Moreover, numerous cohort studies have demonstrated a significant association between childhood obesity and increased risk of both internalizing (e.g., anxiety, depression, somatic problems) and behavioral problems (e.g., aggression, hyperactivity, conduct disorder). In a cross-sectional study of 43,297 children aged 10–17 years, obesity was associated with approximately 60% higher chance of internalizing problems and 30% higher chance of externalizing problems, even after accounting for social and demographic factors [17].

The etiology is complex and likely bidirectional, aligning with models of obesity as both a metabolic [64] and psychosocial disorder [8,68]. Emotional dysregulation, impaired self-esteem and impulsivity may contribute to unhealthy eating patterns and sedentary behaviors, thereby increasing obesity risk [8]. Conversely, obesity may worsen psychological outcomes, through pathophysiological mechanisms of chronic inflammation and subsequent metabolic imbalance, neurovascular dysfunction, and neuroinflammation [8,9,68]. Interestingly, children with attention deficit hyperactivity disorder (ADHD) demonstrate increased prevalence of obesity, reflecting a multifactorial interplay of genetic factors, neurobiological alterations in fronto-striatal and dopaminergic systems, and environmental influences, such as impulsivity and reward-processing abnormalities [69,70].

The lack of parallel findings in the YSR at baseline is noteworthy and may reflect differences between parent-reported and self-reported psychopathology in adolescents, with internalizing symptoms being less likely to be detected by external observers [71,72]. Furthermore, adolescents may have a tendency to underreport or minimize their own emotional and behavioral symptoms, due to limited insight, reluctance to disclose distress, or social desirability concerns [73]. Overall, the value of using multi-informant assessments to obtain a comprehensive understanding of psychosocial functioning is highlighted, as well as the need for integrated prevention and treatment strategies addressing both behavioral and metabolic aspects of pediatric obesity.

### 4.2. Effects of the Lifestyle Intervention on Mental and Cardiometabolic Health

In the present study, a structured, multidisciplinary, lifestyle intervention program implemented for one-year was associated with improvements in social and school competence, psychosocial symptoms, and cardiometabolic outcomes in children and adolescents, with particularly significant benefits among those with excess adiposity. Our findings align with previous studies that demonstrated amelioration of cardiometabolic risk factors and decreased inflammatory markers following the implementation of similar interventions in children, thereby supporting the interrelation between cardiometabolic and psychosocial function [38,63,64,65,66]. Indeed, improvements were observed across multiple domains of psychosocial functioning, most notably in internalizing, externalizing, and total competence scales on the parent-reported CBCL, and in internalizing and total competence symptoms on the self-reported YSR. Overall, the implemented lifestyle modification was associated with improvements on both metabolic balance and mental health. The observed benefits in both quantitative and categorical psychometric score analyses further suggest that the intervention not only reduced symptom severity but also was accompanied by shifts in a proportion of participants from clinically concerning scores into normal functioning. Nevertheless, the observed improvements could also reflect natural maturation, statistical regression to the mean, or the influence of external factors, rather than the intervention alone.

The present findings align with those of multiple meta-analyses [36,37,38,74,75,76,77]. In a meta-analysis of 26 studies (randomized and non-randomized controlled trials), which included 3511 children and adolescents with overweight or obesity, lifestyle interventions produced moderate reduction in depressive symptoms and enhanced quality of life, self-concept and emotional functioning [36]. Another meta-analysis showed reductions in depressive and anxiety symptoms post-intervention, with the former being positively associated with a higher BMI z-score at baseline and the latter with a longer duration of interventions [37]. Similarly, a systematic review covering 73 obesity interventions (n = 6305) found that psychological and multicomponent interventions reduced depressive symptoms and improved health-related quality of life (HRQoL), and that physical activity and multidisciplinary interventions also improved cardiometabolic outcomes [38]. Self-esteem and body image also improve both post-intervention and at medium term [77]. Specifically, physical activity components have been linked to broader benefits on depression, behavioral issues [74], and ADHD [75]. In school-based programs, exercise has been associated with measurable gains in resilience, well-being and positive mental health, and reductions in anxiety [76]. Indeed, in children with obesity acute physical activity may attenuate HPA-axis hyperresponsiveness and support a more adaptive stress response, potentially counteracting chronic stress-related activation of the axis [78,79,80]. The sleep component of interventions, aiming to improve circadian sleep–wake cycle and HPA-axis activity, contributes to BMI-related outcomes in such interventions [81,82]. Finally, mindfulness and stress-management components in pediatric obesity interventions have shown promising results in reducing adiposity markers and stress symptoms [83]. In an 8-week mindfulness program in school-age children with obesity, perceived stress was reduced, ghrelin altered, and BMI improved [84]. In adolescent trials, mental health and excess weight gain improved when mindfulness was paired with diet/physical activity education [85]. The overall evidence suggests that multicomponent interventions with structured diet, physical activity, sleep regularity, and mindfulness skills, yield stronger psychological as well as metabolic and hormonal benefits [33,38,86]. Given that the lifestyle-intervention effects in youth often attenuate over time [87] and maintaining long-term change is challenging [88], follow-up beyond one year is essential to confirm that metabolic and psychosocial benefits persist over time [89].

### 4.3. Adiposity, Inflammation, and Brain Function

The regression analyses in the present study identified multiple anthropometric, metabolic, and endocrine parameters as significant predictors of changes in psychometric outcomes during the lifestyle intervention, highlighting the biopsychosocial complexity of obesity-related mental health in youth. Indices of visceral adiposity (waist circumference, waist-to-height ratio) and markers of glucose and lipid metabolism (triglycerides, HDL-c, glucose, insulin, HbA1C) emerged as consistent predictors of psychosocial score reduction across several CBCL and YSR scales.

The mechanisms underpinning the concurrent psychosocial and metabolic improvements are most likely multifactorial and follow a bidirectional model [9]. From a biological perspective, fat accumulation results in a metabolic imbalance (dyslipidemia, insulin and leptin resistance, gut dysbiosis, hypertension, HPA axis dysregulation) and induces a chronic low-grade inflammatory state, marked by elevated pro- and anti-inflammatory cytokines (e.g., IL-6, TNF), vascular dysfunction, and subsequent neuroinflammation, which can impair brain function and act as a shared biological pathway between metabolic, psychological, and neurocognitive impairment [9,37,90,91,92]. In two longitudinal cohorts of 4835 adolescents and young adults, aged 10–28 years, and 39,613 adults, aged 39–86 years, higher baseline IL-6 concentrations were associated with more adverse depressive symptomatology, with the strongest associations presenting across adolescence, supporting a potential role of systemic inflammation in shaping the severity and progression of mood difficulties [93]. In another study of clinically depressed adolescents, TNF-α was associated with parent-reported depression severity [94]. Moreover, a meta-analysis of 20,791 children and adolescents associated concurrent depression with CRP and IL-6, while depression proved a significant predictor of IL-6 [95].

Reductions in visceral adiposity through lifestyle changes provide a more favorable metabolic and hormonal profile, decreasing chronic inflammation [9]. Consequently, enhanced neurocognitive functioning and mood regulation is promoted via amelioration of neuroinflammation and neurotransmitter signaling, such as in the serotonergic pathways, and modulation of the HPA axis and brain-derived neurotrophic factor (BDNF) expression [9,77,92,96]. Interestingly, exercise-induced increases in BDNF may enhance synaptic plasticity, thereby improving emotional regulation and executive functioning [77]. Psychosocially, structured engagement in physical activity, nutrition education and group-based activities can foster self-efficacy, and improve self-esteem, body image and positive peer interactions, which in turn reduce emotional distress and maladaptive behaviors [36,37,38,74,75,76,77]. Interestingly, a meta-analysis of 50 lifestyle programs found that combining physical activity/exercise with dietary/nutritional changes is likely to lower CRP, IL-6, and IL-1β concentrations, and may also reduce IL-8 in healthy children and adolescents who have overweight or obesity [34]. Meta-analysis data also associate psychological interventions with reduction of pro-inflammatory biomarkers [97]. A lifestyle intervention in children with obesity also showed reduced systemic inflammation and improved mental health, alongside meaningful shifts in gut microbiota composition [98]. Another obesity lifestyle intervention in 594 youth indicated that emotional eating, external eating, and attachment anxiety were associated with increased CRP concentrations, and that while the lifestyle intervention successfully lowered inflammation, the extent of this improvement was diminished in participants reporting higher attachment avoidance [99].

### 4.4. Stress and the Hypothalamic–Pituitary–Adrenal (HPA) Axis

Endocrine parameters emerged as key biological predictors of symptom improvement—highlighting how stress system activity, pubertal maturation, and thyroid function may influence mental health effects in children with obesity. Importantly, in the present one-year lifestyle intervention, the reduction in ACTH and cortisol concentrations, which reflects a normalization of the HPA axis function, was associated with problem attenuation in multiple internalizing, externalizing and behavior domains. On the other hand, the increase in DHEAS correlated negatively with affective and attention problem score reduction.

In obesity, stress physiology plays a multifaceted pathogenic role, contributing to both metabolic dysregulation and neurobehavioral dysfunction [100]. The stress system, consisting of the HPA axis and the sympathetic–adrenal system, coordinates metabolic, behavioral, and autonomic responses to support adaptation and maintain homeostasis [20,22]. In obesity, persistent CRH- and vasopressin-mediated ACTH secretion may lead to chronic mild hypercortisolism and a state of allostatic overload in some individuals [20]. Metabolism redirects towards energy conservation, promoting visceral adiposity, insulin resistance, dyslipidemia, and hypertension, while suppressing anabolic processes, such as growth, thyroid, and reproductive functions [9,100,101]. Moreover, the expansion of visceral adipose tissue can further reinforce stress activation by releasing inflammatory mediators that stimulate the HPA axis [9,21,22]. A stress-related shift of energy sources from growth to survival takes place, metabolic adaptations paralleled by neurobehavioral effects [21]. During the neurodevelopmentally sensitive period of childhood, chronic glucocorticoid exposure may disrupt amygdala, hippocampus, and prefrontal cortex maturation, contributing to difficulties in emotional regulation, executive functioning, and reward sensitivity [22,102]. Stress-responsive neural adaptations in circuits involved in arousal, reward processing, and fear responses may take place, reducing inhibitory control over food intake and impulsive behaviors [20,21]. Indeed, palatable food seems to enhance cortisol production, while hypercortisolism further stimulates comfort eating, which relieves negative mood but promotes visceral fat accumulation via local glucocorticoid signaling [103,104]. The expansion of visceral fat increases the release of proinflammatory cytokines, which in turn stimulate the HPA axis, creating a self-reinforcing cycle of weight gain, metabolic dysfunction, and psychological impairment [9,22,101,105].

Importantly, HPA dysregulation may manifest without elevated basal cortisol concentrations. In some individuals with obesity, tissue-specific glucocorticoid action (e.g., via 11β-HSD1) and alterations in diurnal rhythmicity—especially evening cortisol elevations—further promote central fat accumulation [21,22,100,106]. Chronic stress can also produce glucocorticoid receptor resistance, weakening cortisol’s anti-inflammatory feedback and sustaining a low-grade inflammation linked to mood and cognitive impairment [100]. However, interindividual differences in glucocorticoid sensitivity are observed, with some subjects with obesity showing normal or even low cortisol concentrations, such as in melancholic depression, which are influenced by genetic and receptor-level variations. Furthermore, the adrenal androgen DHEAS influences NMDA and GABAergic signaling, and although it is generally neuroprotective, may lead to increased stress sensitivity and attention dysregulation when in excess [107]. Lifestyle and psychosocial interventions in youngsters have overall proven successful in regulating cortisol [108], reducing inflammation [109], and alleviating both obesity and internalizing and externalizing symptoms [19,110].

Taken together, these findings indicate that the psychosocial improvements observed in the present intervention are most likely modulated not just by cardiometabolic improvements restoring the chronic inflammatory state of obesity, but by amelioration of stress-system overload and a putative HPA-axis recalibration. This perspective underscores the importance of HPA-axis modulation as a therapeutic target in lifestyle interventions for youth with overweight/obesity [9,100].

### 4.5. Sex Differences

A gender-stratified analysis revealed that several benefits of the lifestyle intervention were predominantly observed in males, with improvements across specific CBCL and YSR subscales (withdrawn-depressed, somatic complaints, though problems, attention problems, aggressive behavior, affective problems, ADHD problems, oppositional defiant problems), while females did not demonstrate comparable changes. On the other hand, significant improvement in the positive quality scale of YSR was only observed in female participants.

Meta-analytic evidence demonstrates that multicomponent childhood obesity interventions are not associated with different anthropometric outcomes between sexes [111], and although such programs are associated with reductions in depressive and anxiety symptoms overall, most trials have not reported sex-stratified mental-health outcomes [37]—highlighting a gap our data help to address. Epidemiological studies consistently show that females with obesity show higher internalizing symptoms, such as depression and anxiety [112,113], and are more exposed to cultural differences, weight-related stigma, body dissatisfaction, and lower self-esteem, all of which can attenuate short-term psychosocial gains in interventions [114]. At the same time, biological gender differences in fat distribution, hormonal influences during puberty, and cardiometabolic responses to lifestyle modification may further contribute to the differential outcomes observed [114,115,116,117]. Early-maturing girls are particularly vulnerable to internalizing problems, possibly due to heightened estradiol-driven sensitivity of limbic circuits, increased social stress, and mismatched development between the emotional and regulatory systems [118,119]. Interestingly, longitudinal data associate higher inflammatory marker concentrations, such as IL-6, at the age of 9 years in females with worse depression symptoms compared to males [93]. In boys, rising testosterone during early pubertal stages has been associated with externalizing behaviors, including aggression and rule-breaking behavior [120], whereas lower testosterone concentrations are associated with depressive symptoms [121,122]. As far as gender differences in engagement and adherence are concerned, boys tend to show higher participation in physical activity during obesity interventions [123,124]. Taken together, these sociocultural, hormonal, and adherence-related mechanisms may explain why males in our study demonstrated more robust psychosocial improvements, suggesting the importance of tailoring intervention strategies to better address the unique psychosocial needs of females with obesity.

### 4.6. Strengths and Limitations

The present study has several notable strengths. First, it is one of the few longitudinal, multidisciplinary interventions examining both metabolic and psychosocial outcomes in a large cohort of children and adolescents with overweight and obesity for a long period of time. By combining multiple anthropometric, cardiometabolic, endocrine, and psychometric assessments, a holistic perspective is provided on metabolic health and emotional/behavioral functioning. Moreover, the identification of specific metabolic and hormonal predictors of psychosocial change adds mechanistic depth and highlights potential biological targets for precision interventions. In addition, the use of both parent-reported (CBCL) and self-reported (YSR) psychometric tools strengthens the validity of behavioral assessments across age groups.

However, several limitations must be acknowledged. Recruitment was performed at a single center, which may limit the generalizability of the findings to other populations and healthcare settings. Factors such as detailed dietary micro- and macro- nutrient intake, physical activity monitoring (e.g., accelerometry), social support, and home environment were not examined in detail. The incorporation of neuroimaging, such as functional MRIs, and inflammatory biomarker analysis (interleukins, TNF-α etc.) could also elucidate the biological mediators associating metabolic health to emotional functioning. Finally, the absence of a non-intervention control group increases the risk of selection bias and limits the ability to draw definitive causal inferences between the intervention and the observed improvements.

Overall, multicenter and multinational cohorts with non-intervention control groups are necessary to examine the applicability of the findings to other populations, cause-and-effect relationships, and decode the pathophysiological mechanisms, which connect metabolic and psychosocial improvements post-intervention. Furthermore, while psychometric gains from the one-year lifestyle intervention were evident, further research is needed to determine the sustainability of these benefits beyond the one-year mark and clarify the relative contribution of biological versus behavioral mechanisms.

## 5. Conclusions

Our study demonstrates that a personalized, multidisciplinary lifestyle intervention program is not only associated with improvements in cardiometabolic health in children and adolescents with overweight and obesity, but could also positively affect psychosocial function. These findings underscore the significant benefits of implementing a lifestyle intervention program of healthy diet, good quality sleep, and regular exercise. These programs have the potential, if integrated into healthcare systems with connection to schools, primary care settings, and community programs, to identify specific metabolic and hormonal predictors of physiological and psychological change. Though the interplay between body composition, endocrine signaling and mental health during development is complex, the presented data suggest that lifestyle interventions may have the potential of affecting the bidirectional cycle between excess adiposity and poor mental health in youth.

## 6. Future Perspectives

Future research should evaluate and validate these findings across real-world, multicenter studies and employ prospective randomized controlled designs to establish causal relationships between personalized lifestyle interventions and both metabolic and psychosocial outcomes. Incorporating comprehensive assessments of inflammatory, hormonal, and neuroimmune biomarkers may further clarify the biological pathways associating changes in adiposity and metabolic health with improvements in emotional and behavioral functioning. The integration of digital monitoring tools—such as wearables, mobile applications, and ecological momentary assessment—could enhance the precision of adherence tracking and provide richer insights into behavioral patterns that influence treatment response. Long-term follow-up is essential to determine whether early improvements translate into durable mental health benefits and reduced cardiometabolic burden into adulthood. In addition, larger multicenter studies are important to strengthen generalizability and to explore potential differences in intervention effects across gender, developmental stage, and socioeconomic background.

## Figures and Tables

**Table 1:** Anthropometric (A), hematologic (B), biochemical (C), cardiometabolic risk factor (D), and hormonal (E) investigations in participants with obesity (N = 236), overweight (N = 182), normal BMI (N = 119), and all subjects (N = 537) at initial and annual assessment.

**A. Anthropometric** **Parameters**	**Initial Assessment**					**Annual Assessment**					***p* Between Timepoints**
	**Obesity**	**Overweight**	**Normal BMI**	**All Groups**	***p* Within Baseline**	**Obesity**	**Overweight**	**Normal BMI**	**All Groups**	***p* Within Follow Up**	
**Age (years)**	10.26 ± 2.37	10.18 ± 2.19	9.86 ± 2.45	10.15 ± 2.79	NS	11.35 ± 2.27	11.01 ± 2.47	10.90 ± 2.15	11.06 ± 2.32	NS	**0.01/0.01/0.01/0.01**
**Body weight (kg)**	62.63 ± 17.26	48.98 ± 13.15 ^#^	38.42 ± 11.31 ^#,+^	52.68 ± 16.92	**0.01**	68.59 ± 18.21	53.25 ± 14.14 ^#^	42.69 ± 10.43 ^#,+^	54.16 ± 17.52	**0.01**	**0.01/0.01/0.01/0.01**
**Height (cm)**	146.76 ± 15.94	143.35 ± 14.03 ^#^	139.40 ± 12.21 ^#,+^	143.99 ± 13.62	**0.01**	153.56 ± 14.49	149.48 ± 13.95 ^#^	145.86 ± 12.41 ^#,+^	149.46 ± 13.94	**0.01**	**0.01/0.01/0.01/0.01**
**BMI (kg/m^2^)**	28.30 ± 3.27	23.30 ± 2.19 ^#^	19.35 ± 2.27 ^#,+^	24.81 ± 5.23	**0.01**	28.51 ± 3.34	23.33 ± 2.31 ^#^	19.75 ± 2.29 ^#^	23.63 ± 4.29	**0.01**	**0.01/0.01/**NS**/0.01**
**BMI z-score**	2.77 ± 0.92	1.29 ± 0.63 ^#^	0.19 ± 0.61 ^#,+^	1.70 ± 1.18	**0.01**	2.44 ± 0.84	1.11 ± 0.50 ^#,+^	0.09 ± 0.64 ^#,+^	1.14 ± 1.11	**0.01**	**0.01/0.01/0.01/0.01**
**SBP (mmHg)**	114.61 ± 10.21	108.30 ± 11.02 ^#^	107.16 ± 8.99 ^#^	110.85 ± 11.54	**0.01**	114.75 ± 10.47	109.15 ± 11.02 ^#^	106.77 ± 9.66 ^#^	109.86 ± 10.86	**0.01**	**0.05/**NS/NS**/0.01**
**DBP (mmHg)**	68.39 ± 8.71	66.07 ± 8.75 ^#^	64.66 ± 9.80 ^#^	66.80 ± 9.03	**0.03**	66.62 ± 8.42	66.10 ± 9.17	64.76 ± 8.20	65.79 ± 8.66	NS	**0.05**/NS/NS**/0.05**
**Waist (cm)**	88.00 ± 11.79	77.01 ± 8.63 ^#^	66.65 ± 7.90 ^#,+^	80.00 ± 13.62	**0.01**	91.87 ± 12.05	78.43 ± 7.87 ^#^	69.40 ± 7.09 ^#,+^	78.87 ± 12.35	**0.01**	NS/NS/**0.05**/NS
**Hip (cm)**	94.48 ± 10.93	84.59 ± 7.82 ^#^	75.66 ± 6.97 ^#,+^	87.61 ± 11.68	**0.01**	97.05 ± 10.88	86.25 ± 8.69 ^#^	79.82 ± 7.88 ^#,+^	86.88 ± 11.18	**0.01**	**0.05**/NS/**0.01/**NS
**Waist to Hip ratio**	0.94 ± 0.06	0.92 ± 0.05 ^#^	0.86 ± 0.04 ^#,+^	0.92 ± 0.05	**0.01**	0.95 ± 0.06	0.91 ± 0.05 ^#^	0.87 ± 0.05 ^#,+^	0.90 ± 0.06	**0.01**	NS/NS/NS/NS
**Waist to Height ratio**	0.60 ± 0.06	0.54 ± 0.02 ^#^	0.48 ± 0.05 ^#,+^	0.55 ± 0.03	**0.01**	0.60 ± 0.05	0.53 ± 0.03 ^#^	0.48 ± 0.03 ^#,+^	0.53 ± 0.06	**0.01**	**0.01/0.01/**NS//**0.01**
**B. Hematologic** **Parameters**	**Initial Assessment**					**Annual Assessment**					***p* Between Timepoints**
	**Obesity**	**Overweight**	**Normal BMI**	**All Groups**	***p* Within Baseline**	**Obesity**	**Overweight**	**Normal BMI**	**All Groups**	***p* Within Follow Up**	
**WBC**	7.41 ± 1.87	7.31 ± 1.80	7.07 ± 2.05	7.30 ± 1.89	NS	7.52 ± 1.77	6.82 ± 1.48 ^#^	6.56 ± 1.93 ^#^	6.93 ± 1.74	**0.01**	NS/**0.01**/NS/**0.01**
**RBC**	5.02 ± 0.41	4.96 ± 0.43	4.91 ± 0.45 ^#^	4.98 ± 0.43	**0.05**	5.05 ± 0.42	4.99 ± 0.45	4.96 ± 0.41	4.99 ± 0.43	NS	NS/NS/NS/NS
**Hb**	12.91 ± 0.90	12.72 ± 0.89 ^#^	12.81 ± 0.80	12.82 ± 0.88	**0.05**	12.93 ± 0.95	12.96 ± 1.06	12.91 ± 0.85	12.92 ± 0.97	NS	**0.05**/NS/NS/**0.05**
**Hct**	40.25 ± 2.57	39.74 ± 2.46 ^#^	40.13 ± 2.34	40.05 ± 2.49	**0.05**	40.39 ± 2.70	40.34 ± 3.10	40.17 ± 2.20	40.30 ± 2.73	NS	NS/NS/NS/NS
**PLT**	298.63 ± 60.73	302.79 ± 65.75	286.83 ± 61.53 ^+^	297.40 ± 62.78	**0.05**	294.70 ± 67.05	286.55 ± 59.93	281.82 ± 56.51	287. 35 ± 60.99	NS	NS/**0.01/0.05/0.01**
**ESR**	18.81 ± 12.24	17.01 ± 10.33	13.14 ± 7.89 ^#,+^	16.91 ± 10.95	**0.01**	18.47 ± 12.07	16.97 ± 11.69	12.56 ± 7.42	16.02 ± 10.91	**0.01**	NS/NS/NS/NS
**Ferritin**	59.94 ± 37.20	52.19 ± 49.75	47.36 ± 30.31 ^#^	54.46 ± 40.68	**0.05**	50.08 ± 26.33	53.51 ± 47.37	43.37 ± 26.72 ^#,+^	49.33 ± 36.72	NS	**0.05**/NS/NS/**0.01**
**Folic Acid**	11.00 ± 7.34	10.15 ± 4.45	10.82 ± 4.71	10.67 ± 5.95	NS	7.36 ± 4.33	8.19 ± 4.45	8.97 ± 4.21 ^#^	8.22 ± 4.37	**0.01**	**0.01/0.01/0.01/0.01**
**C. Biochemical** **Parameters**	**Initial Assessment**					**Annual Assessment**					***p* Between Timepoints**
	**Obesity**	**Overweight**	**Normal BMI**	**All Groups**	***p* Within Baseline**	**Obesity**	**Overweight**	**Normal BMI**	**All Groups**	***p* Within Follow Up**	
**Urea**	28.03 ± 6.48	28.48 ± 6.51	28.68 ± 5.39	28.33 ± 6.25	NS	28.77 ± 5.94	28.56 ± 6.55	27.92 ± 6.07	28.74 ± 6.23	NS	NS/NS/NS/NS
**Creatinine**	0.51 ± 0.10	0.51 ± 0.11	0.48 ± 0.11 ^#^	0.50 ± 0.11	**0.05**	0.51 ± 0.10	0.50 ± 0.11	0.48 ± 0.10 ^#^	0.52 ± 0.10	**0.05**	**0.01/0.01/0.01/0.01**
**Uric Acid**	4.82 ± 1.04	4.44 ± 0.88 ^#^	3.97 ± 0.81 ^#,+^	4.50 ± 0.99	**0.01**	4.84 ± 0.79	4.53 ± 0.97 ^#^	4.07 ± 1.02 ^#,+^	4.43 ± 0.98	**0.01**	**0.05**/NS/NS/NS
**K**	4.36 ± 0.25	4.39 ± 0.26	4.38 ± 0.30	4.38 ± 0.27	NS	4.37 ± 0.24	4.40 ± 0.27	4.37 ± 0.27	4.38 ± 0.26	NS	NS/NS/NS/NS
**Na**	140.17 ± 1.40	140.03 ± 1.43	140.46 ± 1.55 ^+^	140.19 ± 1.45	**0.05**	139.90 ± 1.42	139.97 ± 1.49	140.29 ± 1.30 ^#^	140.20 ± 1.42	**0.05**	NS/**0.05/**NS/**0.01**
**Cl**	101.45 ± 2.36	101.80 ± 2.36	102.57 ± 1.99 ^#^	101.79 ± 2.32	**0.05**	101.44 ± 2.24	101.55 ± 2.56	102.55 ± 1.83 ^#,+^	101.08 ± 2.29	**0.05**	**0.01**/NS/NS/**0.01**
**AST**	22.74 ± 5.16	23.16 ± 6.33	23.73 ± 5.52	23.11 ± 5.66	NS	23.49 ± 5.25	23.30 ± 6.09	23.39 ± 5.31	23.40 ± 5.61	NS	**0.01/0.01/**NS**/0.01**
**ALT**	21.06 ± 9.25	18.30 ± 8.29 ^#^	15.77 ± 4.48 ^#,+^	18.94 ± 8.32	**0.01**	22.89 ± 9.98	18.91 ± 7.90 ^#^	16.16 ± 4.75 ^#,+^	18.95 ± 8.18	**0.01**	NS/**0.05**/NS/NS
**γ-GT**	14.81 ± 0.35	12.54 ± 0.35 ^#^	10.91 ± 0.34 ^#,+^	13.17 ± 4.94	**0.01**	16.21 ± 6.76	12.94 ± 3.83 ^#^	11.32 ± 3.58 ^#,+^	12.57 ± 5.14	**0.01**	**0.01**/NS/NS/**0.05**
**Albumin**	4.62 ± 0.21	4.62 ± 0.24	4.63 ± 0.20	4.62 ± 0.22	NS	4.61 ± 0.20	4.64 ± 0.25	4.62 ± 0.19	4.58 ± 0.22	NS	**0.05**/NS/NS/NS
**ALP**	237.54 ± 76.52	223.87 ± 77.25	243.09 ± 69.20 ^+^	234.24 ± 75.50	**0.05**	248.33 ± 76.87	229.82 ± 75.57 ^#^	240.75 ± 68.23	240.45 ± 73.94	**0.05**	**0.01**/NS/NS/**0.01**
**P**	4.60 ± 0.45	4.69 ± 0.59	4.69 ± 0.51	4.65 ± 0.53	NS	4.66 ± 0.52	4.68 ± 0.55	4.68 ± 0.46	4.66 ± 0.51	NS	NS/NS/NS/NS
**Ca**	9.85 ± 0.33	9.77 ± 0.32 ^#^	9.76 ± 0.31 ^#^	9.81 ± 0.33	**0.05**	9.86 ± 0.35	9.82 ± 0.32	9.78 ± 0.31	9.76 ± 0.33	NS	NS/NS/NS/**0.05**
**D. Cardiometabolic Risk Factors**	**Initial Assessment**					**Annual Assessment**					***p* Between Timepoints**
	**Obesity**	**Overweight**	**Normal BMI**	**All Groups**	***p* Within Baseline**	**Obesity**	**Overweight**	**Normal BMI**	**All Groups**	***p* Within Follow Up**	
**Total Cholesterol**	153.71 ± 24.04	159.92 ± 27.55 ^#^	155.86 ± 25.58	156.27 ± 25.70	**0.05**	153.89 ± 19.13	154.59 ± 23.93	150.41 ± 18.71	153.11 ± 21.17	NS	NS/NS/NS/NS
**Triglycerides**	78.88 ± 39.31	75.99 ± 72.05	62.06 ± 34.49 ^#,+^	74.10 ± 52.11	**0.05**	92.04 ± 43.59	72.87 ± 39.76 ^#^	60.89 ± 22.19 ^#,+^	74.46 ± 38.34	**0.01**	NS/NS/NS/NS
**HDL-c**	49.77 ± 10.55	54.96 ± 11.41 ^#^	59.78 ± 14.53 ^#,+^	53.77 ± 12.46	**0.01**	51.05 ± 11.89	55.99 ± 11.30 ^#^	60.18 ± 12.89 ^#,+^	55.92 ± 12.43	**0.01**	**0.01/0.01**/NS/**0.01**
**LDL-c**	88.45 ± 21.49	91.12 ± 23.80	84.22 ± 20.72 ^+^	88.38 ± 22.22	**0.01**	84.69 ± 16.89	84.94 ± 21.46	78.39 ± 16.82 ^#,+^	82.86 ± 19.10	**0.05**	**0.01/0.01/**NS**/0.01**
**ApoA1**	134.47 ± 19.14	141.94 ± 17.71 ^#^	147.35 ± 22.79 ^#,+^	139.89 ± 20.21	**0.05**	137.11 ± 19.10	140.21 ± 18.10	143.70 ± 21.00 ^#^	140.44 ± 19.40	**0.05**	NS/NS/NS/NS
**ApoB**	75.39 ± 16.01	75.82 ± 16.38	70.99 ± 13.87 ^#,+^	74.54 ± 15.77	**0.05**	75.46 ± 13.59	73.95 ± 13.96 #	68.94 ± 12.18 ^#,+^	72.82 ± 13.56	**0.01**	NS/NS/**0.05**/NS
**Lp(a)**	17.01 ± 29.14	16.7 ± 22.76	16.9 ± 25.94	17.01 ± 26.37	NS	17.93 ± 25.40	19.86 ± 27.40	13.17 ± 20.04 ^+^	17.29 ± 24.90	**0.05**	NS/NS/NS/NS
**Glucose**	81.56 ± 6.95	78.98 ± 7.38 ^#^	79.37 ± 7.11 ^#^	80.00 ± 7.22	**0.01**	84.16 ± 6.24	82.08 ± 6.83 ^#^	80.61 ± 7.40 ^#^	82.16 ± 6.97	**0.05**	**0.01/0.01/0.01/0.01**
**HbA1C**	5.24 ± 0.24	5.22 ± 0.22	5.12 ± 0.25 ^#,+^	5.21 ± 0.24	**0.01**	5.23 ± 0.22	5.15 ± 0.24 ^#^	5.10 ± 0.23 ^#^	5.16 ± 0.23	**0.01**	**0.01/**NS/NS**/0.01**
**HbA1**	6.1 ± 0.40	6.07 ± 0.39	5.9 ± 0.40 ^#,+^	6.05 ± 0.41	**0.01**	6.19 ± 0.36	6.06 ± 0.35 ^#^	5.94 ± 0.34 ^#,+^	6.06 ± 0.36	**0.05**	NS/**0.05**/NS**/0.05**
**HOMA-IR**	3.71 ± 2.60	2.36 ± 1.52 ^#^	1.81 ± 1.02 ^#,+^	2.83 ± 2.15	**0.01**	4.22 ± 2.46	2.59 ± 1.36 ^#^	2.26 ± 1.47 ^#^	2.93 ± 1.92	**0.01**	NS/**0.05/0.05/0.05**
**E. Hormonal** **Parameters**	**Initial Assessment**					**Annual Assessment**					
	**Obesity**	**Overweight**	**Normal BMI**	**All Groups**	***p* Within Baseline**	**Obesity**	**Overweight**	**Normal BMI**	**All Groups**	***p* Within Follow Up**	***p* Between Timepoints**
**TSH**	3.07 ± 1.68	2.88 ± 1.43	2.78 ± 1.35	2.94 ± 1.53	NS	3.18 ± 1.47	2.78 ± 1.44 ^#^	2.63 ± 1.09 ^#^	2.84 ± 1.36	**0.01**	NS/NS/NS/NS
**FT4**	1.12 ± 0.14	1.12 ± 0.14	1.13 ± 0.13	1.12 ± 0.14	NS	1.07 ± 0.13	1.08 ± 0.16	1.07 ± 0.14	1.07 ± 0.15	NS	**0.01/0.01/0.01/0.01**
**T3**	146.15 ± 27.04	139.14 ± 24.59 ^#^	132.77 ± 26.37 ^#,+^	140.77 ± 26.55	**0.05**	137.19 ± 23.92	129.94 ± 28.84 ^#^	130.87 ± 21.71	132.16 ± 25.61	**0.05**	**0.01/0.01/**NS**/0.01**
**AntiTG**	33.20 ± 130.86	28.48 ± 48.77	28.11 ± 48.07	30.45 ± 93.60	NS	23.43 ± 26.52	27.55 ± 46.55	27.50 ± 37.86	26.41 ± 39.22	NS	NS/NS/NS/**0.05**
**AntiTPO**	29.33 ± 103.06	28.02 ± 110.73	45.29 ± 171.85	32.50 ± 124.20	NS	24.29 ± 103.36	24.84 ± 86.37	43.68 ± 166.78	30.51 ± 120.86	NS	NS/NS/NS/NS
**IGF-I**	280.96 ± 169.49	283.04 ± 160.90	271.45 ± 159.49	279.54 ± 160.15	NS	350.32 ± 204.90	319.58 ± 168.40	317.17 ± 152.49	327.25 ± 174.71	NS	**0.01**/NS/NS/**0.01**
**IGFBP-3**	5.06 ± 1.00	4.93 ± 0.92	4.71 ± 0.86 ^#^	4.94 ± 0.95	**0.01**	5.23 ± 0.93	4.98 ± 0.87	4.89 ± 0.99 ^#^	5.02 ± 0.93	**0.01**	**NS**/NS/NS/NS
**Δ4**	0.89 ± 0.78	0.95 ± 1.04	0.84 ± 0.81	0.90 ± 0.88	NS	1.14 ± 0.95	1.18 ± 1.03	1.12 ± 0.87	1.15 ± 0.96	NS	**0.01/0.01/0.01/0.01**
**Testosterone**	45.67 ± 72.83	40.25 ± 69.63	37.90 ± 79.01	42.09 ± 73.16	NS	69.21 ± 99.73	71.57 ± 116.38	46.78 ± 76.83	63.31 ± 101.49	NS	**0.01/0.01**/NS**/0.01**
**DHEAS**	119.90 ± 81.26	101.32 ± 80.72 ^#^	76.96 ± 66.41 ^#,+^	103.95 ± 79.58	**0.01**	139.59 ± 91.34	124.26 ± 80.98	94.31 ± 68.02 ^#,+^	119.25 ± 82.01	**0.01**	**0.01/0.01/0.01/0.01**
**Prolactin**	12.64 ± 7.13	11.70 ± 5.48	12.84 ± 8.59	12.37 ± 7.00	NS	13.32 ± 6.86	13.02 ± 7.45	12.96 ± 7.68	13.08 ± 7.35	NS	**0.05**/NS/**0.05/0.01**
**LH**	1.97 ± 4.07	1.98 ± 3.69	1.56 ± 3.09	1.88 ± 3.74	NS	2.57 ± 3.65	3.11 ± 6.49	2.51 ± 2.93	2.78 ± 4.9	NS	**0.01/0.05/0.05/0.01**
**FSH**	2.38 ± 2.04	2.40 ± 1.97	2.62 ± 1.84	2.44 ± 1.97	NS	2.63 ± 1.86	3.11 ± 2.30	3.62 ± 2.27 ^#^	3.13 ± 2.21	**0.01**	**0.01/0.01/0.01/0.01**
**Ε2**	11.13 ± 16.35	19.86 ± 44.88 ^#^	14.37 ± 30.80	14.79 ± 31.89	**0.01**	19.03 ± 31.84	19.12 ± 34.51	17.95 ± 31.30	18.74 ± 32.74	NS	**0.01**/NS/NS/NS
**ACTH**	31.20 ± 21.96	26.94 ± 22.67 ^#^	25.18 ± 14.75 ^#^	28.40 ± 20.93	**0.05**	34.00 ± 31.12	27.51 ± 20.02	29.06 ± 23.93	29.78 ± 24.77	NS	NS/NS**/0.05**/NS
**Cortisol**	13.21 ± 5.84	13.01 ± 5.18	13.97 ± 6.08	13.31 ± 5.68	NS	12.51 ± 4.78	12.59 ± 4.99	12.18 ± 4.42	12.44 ± 4.75	NS	NS/**0.05**/NS/**0.01**
**PTH**	35.00 ± 11.00	34.97 ± 14.25	37.69 ± 16.05	35.58 ± 13.39	NS	33.88 ± 11.58	35.01 ± 13.18	36.13 ± 13.56	35.07 ± 12.90	NS	NS/NS/NS/NS
**25OHVitD**	23.73 ± 9.57	25.83 ± 9.59 ^#^	27.83 ± 10.43 ^#^	25.35 ± 9.89	**0.05**	24.26 ± 8.71	26.87 ± 9.10	29.49 ± 9.76 ^#,+^	26.99 ± 9.39	**0.05**	NS/NS/NS/NS
**Insulin**	18.15 ± 11.65	12.27 ± 7.09 ^#^	9.09 ± 4.72 ^#,+^	14.13 ± 9.75	**0.01**	20.12 ± 11.59	12.71 ± 6.37 ^#^	11.29 ± 7.23 ^#^	14.28 ± 9.07	**0.01**	NS/NS/**0.05**/NS
**SHBG**	44.16 ± 24.75	60.04 ± 34.74 ^#^	91.0 ± 46.16 ^#,+^	60.53 ± 38.70	**0.01**	41.48 ± 22.79	60.39 ± 37.13 ^#^	79.36 ± 40.69 ^#,+^	61.15 ± 37.86	**0.01**	NS/NS/NS/NS

All results are presented as mean ± standard deviation. Subjects were classified as obese, overweight, or with normal BMI according to IOTF criteria at initial assessment. Tables present comparisons among three groups at both initial and annual assessment. All measured variables were compared by employing repeated-measures ANOVA. Significant main effects were revealed by LSD post hoc test. Statistical significance was set at (*p* < 0.05, rounded to 0.05 in Table), while strong significance (*p* < 0.01, rounded to 0.01 in Table) is also noted. NS: nonsignificant (*p* > 0.05) difference. +: significant difference from Overweight group, #: significant difference from Obese group. *p*-values between two timepoints refer to obese, overweight, and normal BMI respectively. Statistically significant *p*-values appear in bold. ACTH: adrenocorticotropic hormone; ALP: alkaline phosphatase; ALT: alanine transaminase; anti-TG: antibodies against thyroglobulin; anti-TPO: thyroid peroxidase antibodies; ApoA1: apolipoprotein A1; ApoB: apolipoprotein B; AST: aspartate aminotransferase; BMI: body mass index; BW: body weight; DBP: diastolic blood pressure; Δ4: androstenedione; DHEA-s: dehydroepiandrosterone sulfate; E2: estradiol; FSH: follicle stimulating hormone; FT4: free thyroxine; γGT: gamma-glutamyl transferase; HbA1C: hemoglobin A1C; HDL: high-density lipoprotein; HOMA-IR: homeostatic model assessment for insulin resistance; IGF-I: insulin-like growth factor I; LDL: low-density lipoprotein; LH: luteinizing hormone; Lp(a): lipoprotein a; PTH: parathormone; SBP: systolic blood pressure; T3: triiodothyronine; TSH: thyroid stimulating hormone; 25OHD: total 25-OH-vitamin D.

**Table 2:** Psychometric scores in CBCL scales in subjects with obesity (N = 236), overweight (N = 182), normal BMI (N = 119), and all subjects (N = 537) at initial and annual assessment.

CBCL	Initial Assessment					Annual Assessment					*p* Between Timepoints
	Obesity	Overweight	Normal BMI	All Groups	*p* Within Baseline	Obesity	Overweight	Normal BMI	All Groups	*p* Within Follow Up	
**Activities**	22.98 ± 22.72	25.15 ± 23.48	26.55 ± 26.19	24.49 ± 23.76	NS	23.47 ± 24.59	23.47 ± 21.19	24.94 ± 22.98	23.95 ± 22.71	NS	NS/NS/NS/NS
**Social**	33.57 ± 25.92	38.17 ± 23.24	41.44 ± 26.76 ^#^	36.93 ± 25.38	**0.01**	37.98 ± 24.87	40.52 ± 25.49	40.46 ± 23.63	39.79 ± 24.68	NS	**0.01**/NS/NS/**0.01**
**School**	50.88 ± 21.85	55.55 ± 19.79 ^#^	54.41 ± 19.98	53.28 ± 20.81	**0.05**	53.90 ± 21.02	55.84 ± 17.08	59.51 ± 16.81 ^#^	56.45 ± 18.29	**0.05**	**0.05**/NS/**0.05/0.01**
**Total Competence**	26.65 ± 24.39	32.22 ± 24.84 ^#^	33.92 ± 27.66 ^#^	30.24 ± 25.47	**0.05**	29.29 ± 24.95	32.67 ± 25.35	33.88 ± 25.46	32.09 ± 25.27	NS	NS/NS/NS/NS
**Anxious-Depressed**	71.40 ± 17.80	68.59 ± 17.69	66.04 ± 16.75 ^#^	69.27 ± 17.63	**0.01**	66.65 ± 17.63	66.33 ± 16.63	64.61 ± 16.26	65.90 ± 16.79	NS	**0.01/0.01**/NS/**0.01**
**Withdrawn-Depressed**	71.44 ± 18.58	67.22 ± 17.43 ^#^	64.77 ± 16.26 ^#^	68.55 ± 17.88	**0.01**	71.54 ± 18.59	65.65 ± 16.12 ^#^	63.03 ± 15.85 ^#^	66.55 ± 17.09	**0.01**	**0.05/0.05**/NS/**0.01**
**Somatic** **Complaints**	69.60 ± 16.93	68.30 ± 17.32	65.77 ± 16.37 ^#^	68.32 ± 16.97	**0.05**	66.53 ± 17.22	66.03 ± 15.68	62.91 ± 15.58	65.22 ± 16.15	NS	**0.01/0.01**/NS/**0.01**
**Social Problems**	72.29 ± 16.73	67.85 ± 16.00 ^#^	65.03 ± 14.79 ^#^	69.20 ± 16.31	**0.01**	68.88 ± 17.46	62.45 ± 15.29 ^#^	65.22 ± 14.58 ^#^	65.34 ± 15.90	**0.05**	**0.01/0.01/0.05/0.01**
**Though Problems**	65.04 ± 16.99	63.99 ± 16.70	61.50 ± 15.14	63.90 ± 16.52	NS	64.62 ± 17.88	61.49 ± 17.44	61.70 ± 15.24	62.46 ± 16.15	NS	**0.05/0.01**/NS/**0.01**
**Attention** **Problems**	64.08 ± 14.64	61.97 ± 13.25	61.88 ± 13.35	62.88 ± 13.92	NS	64.11 ± 15.17	59.87 ± 12.69 ^#^	61.44 ± 13.48	61.57 ± 13.76	**0.01**	NS/**0.05**/NS/**0.01**
**Rule Breaking** **Behavior**	65.71 ± 15.85	64.42 ± 15.76	64.47 ± 14.38	65.01 ± 15.49	NS	63.71 ± 15.06	62.44 ± 14.68	60.18 ± 12.73	62.12 ± 14.26	NS	**0.01/0.01/0.01/0.01**
**Aggressive** **Behavior**	67.07 ± 17.89	65.52 ± 17.40	61.86 ± 14.13 ^#^	65.40 ± 17.04	**0.01**	65.07 ± 17.58	64.05 ± 16.08	61.49 ± 14.49	63.56 ± 16.09	NS	**0.05/0.01**/NS/**0.01**
**Internalizing Problems**	65.19 ± 29.46	58.73 ± 30.70 ^#^	55.67 ± 28.72 ^#^	60.91 ± 29.93	**0.05**	57.58 ± 32.46	55.41 ± 29.27	52.17 ± 26.85	55.05 ± 29.53	NS	**0.01/0.01**/NS/**0.01**
**Externalizing Problems**	55.64 ± 29.79	53.34 ± 29.23	49.77 ± 28.04	53.57 ± 29.26	NS	50.81 ± 31.25	50.70 ± 28.44	45.77 ± 27.66	49.23 ± 29.07	NS	**0.01/0.01**/NS/**0.01**
**Total Problems**	62.39 ± 29.60	56.35 ± 30.37 ^#^	50.54 ± 28.25 ^#^	57.75 ± 29.88	**0.05**	54.96 ± 32.56	51.09 ± 29.26	45.37 ± 27.54 ^#^	50.47 ± 29.92	**0.01**	**0.01/0.01/0.05/0.01**
**Affective** **Problems**	74.17 ± 18.55	70.39 ± 18.59 ^#^	67.96 ± 18.27 ^#^	71.53 ± 18.27	**0.05**	72.12 ± 18.98	68.39 ± 16.82	66.74 ± 16.79 ^#^	68.97 ± 17.55	**0.05**	**0.01/0.01**/NS/**0.01**
**Anxiety Problems**	73.00 ± 18.09	69.48 ± 18.62	66.89 ± 18.64 ^#^	70.46 ± 18.10	**0.01**	68.45 ± 18.68	66.69 ± 17.41	65.48 ± 17.23	66.83 ± 17.73	NS	**0.01/0.05**/NS/**0.01**
**Somatic Problems**	66.82 ± 18.14	65.82 ± 18.10	64.92 ± 17.25	66.06 ± 17.92	NS	64.65 ± 17.49	65.01 ± 17.08	62.61 ± 16.51	64.17 ± 17.02	NS	**0.05/0.01**/NS/**0.01**
**Attention Deficit Hyperactivity Problems**	63.94 ± 15.98	61.66 ± 14.43	60.77 ± 13.60	62.47 ± 15.00	NS	63.76 ± 15.88	59.77 ± 13.74 ^#^	60.11 ± 13.89	61.03 ± 14.51	**0.05**	**0.05/0.05**/NS/**0.01**
**Oppositional** **Defiant Problems**	65.86 ± 16.18	65.02 ± 15.89	62.16 ± 13.69 ^#^	64.76 ± 15.60	**0.05**	63.96 ± 15.48	64.12 ± 15.03	62.03 ± 13.34	63.44 ± 14.66	NS	**0.01**/NS/NS/**0.01**
**Conduct** **Problems**	64.21 ± 16.37	63.84 ± 16.64	61.59 ± 13.74	63.50 ± 15.92	NS	63.73 ± 16.32	61.67 ± 15.01	58.88 ± 12.72 ^#^	61.42 ± 14.84	**0.01**	**0.05/0.01/0.05/0.01**
**Sluggish** **Cognitive** **Problems**	65.43 ± 17.36	62.20 ± 16.14	60.01 ± 15.07 ^#^	63.15 ± 16.59	**0.01**	63.75 ± 17.55	60.99 ± 15.78	60.72 ± 15.37	61.71 ± 16.20	NS	**0.05**/NS/NS/**0.05**
**Obsessive** **Compulsive** **Problems**	66.75 ± 16.67	65.64 ± 16.75	65.78 ± 15.76	66.16 ± 16.48	NS	64.50 ± 17.01	64.23 ± 14.64	64.26 ± 14.85	64.32 ± 15.38	NS	**0.05/0.05/**NS**/0.01**
**Post Traumatic Stress Problems**	68.95 ± 18.39	66.27 ± 17.68	62.87 ± 15.25 ^#^	66.70 ± 17.63	**0.01**	67.88 ± 18.72	63.50 ± 16.85 ^#^	62.57 ± 15.38 ^#^	64.49 ± 17.09	**0.01**	**0.01/0.05/**NS/**0.01**

All results are presented as mean ± standard deviation. Subjects were classified as obese, overweight, or with normal BMI according to IOTF criteria at initial assessment. Tables present comparisons among three groups at both initial and annual assessment. All measured variables were compared by employing repeated-measures ANOVA. Significant main effects were revealed by LSD post hoc test. Statistical significance was set at (*p* < 0.05, rounded to 0.05 in Table), while strong significance (*p* < 0.01, rounded to 0.01 in Table) is also noted. NS: nonsignificant (*p* > 0.05) difference. #: significant difference from Obese group. *p*-values between two timepoints refer to obese, overweight, and normal BMI respectively. Statistically significant *p*-values appear in bold. BMI: body mass index; CBCL: Child Behavior Checklist.

**Table 3:** Psychometric scores in YSR scales in subjects with obesity (N = 80), overweight (N = 61), normal BMI (N = 28), and all subjects (N = 169) at initial and annual assessment.

YSR	Initial Assessment					Annual Assessment					*p* Between Timepoints
	Obesity	Overweight	Normal BMI	All Groups	*p* Within Baseline	Obesity	Overweight	Normal BMI	All Groups	*p* Within Annual Assessment	
**Activities**	24.10 ± 23.92	26.81 ± 25.95	26.83 ± 26.48	25.53 ± 24.99	NS	18.67 ± 24.54	21.49 ± 21.70	27.33 ± 26.04	22.17 ± 23.93	NS	NS/NS/NS/**0.05**
**Social**	36.19 ± 26.39	44.52 ± 24.52	39.76 ± 27.33	39.84 ± 26.01	NS	36.05 ± 25.76	49.79 ± 24.22 ^#^	44.50 ± 26.47	44.04 ± 25.78	**0.05**	NS/NS/NS/NS
**Total** **Competence**	28.31 ± 25.62	32.26 ± 24.17	31.19 ± 23.31	30.22 ± 24.66	NS	23.30 ± 23.06	32.27 ± 24.26	35.16 ± 26.76 ^#^	30.12 ± 24.84	**0.05**	NS/NS/NS/NS
**Anxious** **Depressed**	67.04 ± 16.68	66.37 ± 17.44	66.10 ± 18.09	66.64 ± 17.10	NS	62.45 ± 15.88	64.74 ± 14.94	63.88 ± 17.59	63.76 ± 15.87	NS	**0.05/**NS/NS/**0.05**
**Withdrawn** **Depressed**	62.34 ± 14.86	64.00 ± 16.51	63.10 ± 17.13	63.07 ± 15.78	NS	64.65 ± 16.13	61.72 ± 15.66	62.06 ± 17.39	62.77 ± 16.20	NS	NS/NS/NS/NS
**Somatic** **Complaints**	63.57 ± 15.07	63.49 ± 15.58	59.52 ± 14.81	62.87 ± 15.20	NS	56.98 ± 10.08	59.84 ± 12.07	60.38 ± 15.44	59.04 ± 12.45	NS	**0.01**/NS/NS/**0.05**
**Social Problems**	66.40 ± 15.29	62.62 ± 15.66	64.69 ± 17.84	64.75 ± 15.87	NS	57.30 ± 12.72	59.76 ± 11.89	58.13 ± 12.21	58.52 ± 12.20	NS	**0.01**/NS/NS/**0.01**
**Though Problems**	60.37 ± 13.24	61.35 ± 13.72	60.83 ± 12.11	60.80 ± 13.17	NS	57.75 ± 12.10	59.90 ± 11.58	57.03 ± 12.04	58.44 ± 11.84	NS	**0.05**/NS/NS/**0.05**
**Attention** **Problems**	61.10 ± 13.24	61.14 ± 13.55	64.41 ± 17.04	61.67 ± 14.02	NS	58.78 ± 14.33	59.88 ± 12.53	57.31 ± 10.50	58.84 ± 12.61	NS	NS/NS/NS/NS
**Rule Breaking Behavior**	58.56 ± 12.59	59.27 ± 14.38	57.10 ± 10.74	58.57 ± 12.95	NS	56.43 ± 8.66	59.50 ± 13.79	54.16 ± 5.87 ^+^	57.09 ± 10.72	**0.05**	NS/NS/NS/NS
**Aggressive** **Behavior**	65.29 ± 15.68	61.57 ± 14.37	63.34 ± 17.01	63.62 ± 15.45	NS	57.70 ± 12.22	63.62 ± 15.80 ^#^	59.09 ± 13.04	60.49 ± 14.14	**0.05**	**0.01**/NS/NS/NS
**Internalizing Problems**	54.11 ± 26.94	50.57 ± 31.95	46.45 ± 32.49	51.55 ± 29.74	NS	45.38 ± 28.04	48.34 ± 26.55	41.78 ± 34.56	45.65 ± 29.19	NS	**0.05**/NS/NS/0.05
**Externalizing Problems**	49.33 ± 26.81	45.24 ± 27.56	45.21 ± 28.73	47.16 ± 27.32	NS	24.84 ± 3.92	49.22 ± 27.07 ^#^	37.63 ± 23.69 ^+^	42.62 ± 25.88	**0.05**	NS/NS/NS/NS
**Total Problems**	52.39 ± 31.19	47.16 ± 30.15	45.52 ± 27.64	49.35 ± 29.15	NS	39.40 ± 26.00	46.62 ± 24.78	36.75 ± 30.30	41.66 ± 26.83	NS	**0.01**/NS/NS**/0.01**
**Affective** **Problems**	69.48 ± 15.12	64.60 ± 17.22	66.28 ± 16.32	67.18 ± 16.17	NS	61.65 ± 13.39	62.06 ± 13.29	59.03 ± 13.47	61.13 ± 13.32	NS	**0.01**/NS/NS**/0.01**
**Anxiety Problems**	64.90 ± 16.71	64.19 ± 16.02	64.38 ± 15.58	64.56 ± 16.19	NS	60.75 ± 13.82	61.78 ± 14.64	63.47 ± 16.27	61.89 ± 14.74	NS	NS/NS/NS/**0.05**
**Somatic Problems**	62.33 ± 14.51	64.17 ± 14.86	58.41 ± 12.85	62.34 ± 14.43	NS	57.03 ± 10.26	61.10 ± 11.87	59.06 ± 14.43	59.23 ± 12.14	NS	**0.05**/NS/NS/NS
**Attention Deficit Hyperactivity Problems**	63.16 ± 13.01	61.48 ± 11.93	63.76 ± 16.23	62.65 ± 13.18	NS	60.55 ± 13.95	58.96 ± 11.05	57.91 ± 11.30	59.20 ± 12.08	NS	NS/**0.05**/NS/NS
**Oppositional** **Defiant Problems**	64.00 ± 14.35	60.71 ± 12.13	64.66 ± 16.24	62.92 ± 13.96	NS	59.15 ± 11.66	63.90 ± 15.69	62.25 ± 15.13	61.91 ± 14.36	NS	NS/NS/NS/NS
**Conduct** **Problems**	60.90 ± 14.64	60.87 ± 15.33	59.41 ± 14.81	60.64 ± 14.85	NS	56.53 ± 9.79	60.16 ± 15.30	54.81 ± 8.34	57.57 ± 12.19	NS	NS/NS/NS/NS
**Obsessive** **Compulsive Problems**	63.28 ± 15.50	65.13 ± 16.12	66.00 ± 15.79	64.40 ± 15.72	NS	63.43 ± 17.55	64.08 ± 15.05	62.03 ± 15.66	63.33 ± 15.95	NS	NS/NS/NS/NS
**Post Traumatic Stress Problems**	63.04 ± 14.56	63.16 ± 16.83	64.79 ± 17.25	63.37 ± 15.79	NS	60.33 ± 14.96	62.02 ± 14.53	61.38 ± 16.23	61.30 ± 15.02	NS	NS/NS/NS/NS
**Positive Qualities**	57.59 ± 28.07	60.92 ± 27.56	62.59 ± 26.53	59.63 ± 27.55	NS	61.03 ± 25.78	62.92 ± 27.06	67.03 ± 26.31	63.38 ± 26.33	NS	NS/NS/NS/NS

All results are presented as mean ± standard deviation. Subjects were classified as obese, overweight, or with normal BMI according to IOTF criteria at initial assessment. Tables present comparisons among three groups at both initial and annual assessment. All measured variables were compared by employing repeated-measures ANOVA. Significant main effects were revealed by LSD post hoc test. Statistical significance was set at (*p* < 0.05, rounded to 0.05 in Table), while strong significance (*p* < 0.01, rounded to 0.01 in Table) is also noted. NS: nonsignificant (*p* > 0.05) difference. +: significant difference from Overweight group, #: significant difference from Obese group. *p*-values between two timepoints refer to obese, overweight, and normal BMI respectively. Statistically significant *p*-values appear in bold. BMI: body mass index; YSR: Youth Self Report.

## Data Availability

The data supporting this study are available from the corresponding author upon request. The data are not publicly accessible due to privacy constraints.

## Supplementary Materials

The following supporting information can be downloaded at: https://www.mdpi.com/article/10.3390/nu18010150/s1, Table S1: Normal and borderline-clinical CBCL scores at initial assessment in subjects with obesity, overweight, and normal BMI; Table S2: Normal and borderline-clinical YSR scores at initial assessment in subjects with obesity, overweight, and normal BMI; Table S3: Comparison of normal and borderline/clinical CBCL scores between initial and annual assessment in subjects with obesity, overweight, normal BMI, and all sample; Table S4: Comparison of normal and borderline/clinical YSR scores between initial and annual assessment in subjects with obesity, overweight, normal BMI, and all sample; Table S5: Psychometric scores categorized by gender in CBCL in subjects with obesity, overweight, normal BMI, and all subjects at initial and annual assessment; Table S6: Psychometric scores categorized by gender in YSR in subjects with obesity, overweight, normal BMI, and all subjects at initial and annual assessment; Table S7: Predictors of psychometric score change after lifestyle intervention in CBCL; Table S8: Predictors of psychometric score change after lifestyle intervention in YSR.
