# Supplementary material for: Parallel Improvement of Both Mental and Cardiometabolic Health in Children and Adolescents with Overweight and Obesity, Following the Implementation of a Multidisciplinary Lifestyle Intervention Program
Source: Nutrients. 2026 Jan 1;18(1):150. doi: 10.3390/nu18010150 (PMC12787964; doi:10.3390/nu18010150)
Supplement: Supplementary file 1 [file nutrients-18-00150-s001.zip › nutrients-4027774-supplementary.pdf]

Supplemental Table S1. Normal and borderline-clinical CBCL scores at initial assessment in subjects with obesity, overweight and normal BMI.

CBCL Scales Initial Assessment	SCORE	OBESITY	OVERWEIGHT	NORMAL BMI	X^2	P-value
**ACTIVITIES**	Normal	159 (41.8%)	131 (34.5%)	90 (23.7%)	3.41	NS
	Borderline-Clinical	78 (49.7%)	51 (32.5%)	28 (17.8%)		
**SOCIAL**	Normal	193 (42.3%)	161 (35.3%)	102 (22.4%)	4.24	NS
	Borderline-Clinical	44 (54.3%)	21 (25.9%)	16 (19.8 %)		
**SCHOOL**	Normal	226 (44.0%)	175 (34.0%)	113 (22.0%)	0.16	NS
	Borderline-Clinical	11 (47.8%)	7 (30.4%)	5 (21.7%)		
**TOTAL COMPETENCE**	Normal	140 (41.1%)	122 (35.8%)	79 (23.2%)	3.59	NS
	Borderline-Clinical	97 (49.5%)	60 (30.6%)	39 (19.9%)		
**ANXIOUS-DEPRESSED**	Normal	185 (42.4%)	152 (34.9%)	99 (22.7%)	2.73	NS
	Borderline-Clinical	52 (51.5%)	30 (29.7%)	19 (18.8%)		
**WITHDRAWN-DEPRESSED**	Normal	175 (40.9%)	150 (35.0%)	103 (24.1%)	10.06	**0.01**
	Borderline-Clinical	62 (56.9%)	32 (29.4%)	15 (13.8%)		
**SOMATIC COMPLAINTS**	Normal	203 (44.0%)	153 (33.2%)	105 (22.8%)	1.44	NS
	Borderline-Clinical	34 (44.7%)	29 (38.2%)	13 (17.1%)		
**SOCIAL PROBLEMS**	Normal	192 (42.4%)	151 (33.3%)	110 (24.3%)	9.30	**0.05**
	Borderline-Clinical	45 (53.6%)	31 (36.9%)	8 (9.5%)		
**THOUGHT PROBLEMS**	Normal	200 (43.5%)	154 (33.5%)	106 (23.0%)	2.15	NS
	Borderline-Clinical	37 (48.1%)	28 (36.4%)	12 (15.6%)		
**ATTENTION PROBLEMS**	Normal	210 (43.0%)	167 (34.2%)	111 (22.7%)	3.09	NS
	Borderline-Clinical	27 (55.1%)	15 (30.6%)	7 (14.3%)		
**RULE BREAKING BEHAVIOUR**	Normal	215 (44.6%)	155 (32.2%)	112 (23.2%)	7.29	**0.05**
	Borderline-Clinical	22 (40.0%)	27 (49.1%)	6 (10.9%)		
**AGGRESSIVE BEHAVIOUR**	Normal	194 (42.6%)	152 (33.4%)	109 (24.0%)	7.05	**0.05**
	Borderline-Clinical	43 (52.4%)	30 (36.6%)	9 (11.0%)		

**INTERNALIZING PROBLEMS**	Normal	141 (41.2%)	116 (33.9%)	85 (24.9%)	5.36	NS
	Borderline-Clinical	96 (49.2%)	66 (33.8%)	33 (16.9%)		
**EXTERNALIZING PROBLEMS**	Normal	167 (41.6%)	133 (33.2%)	101 (25.2%)	9.91	**0.01**
	Borderline-Clinical	70 (51.5%)	49 (36.0%)	17 (12.5%)		
**TOTAL PROBLEMS**	Normal	148 (40.1%)	126 (34.1%)	95 (25.7%)	11.99	**0.01**
	Borderline-Clinical	89 (53.0%)	56 (33.3%)	23 (13.7%)		
**AFFECTIVE PROBLEMS**	Normal	168 (41.6%)	143 (35.4%)	93 (23.0%)	4.30	NS
	Borderline-Clinical	69 (51.9%)	39 (29.3%)	25 (18.8%)		
**ANXIETY PROBLEMS**	Normal	176 (42.5%)	135 (32.6%)	103 (24.9%)	8.90	**0.05**
	Borderline-Clinical	61 (49.6%)	47 (38.2%)	15 (12.2%)		
**SOMATIC PROBLEMS**	Normal	202 (43.8%)	153 (33.2%)	106 (23.0%)	2.09	NS
	Borderline-Clinical	35 (46.1%)	29 (38.2%)	12 (15.8%)		
**ATTENTION DEFICIT-HYPERACTIVITY PROBLEMS**	Normal	209 (43.0%)	165 (35.0%)	112 (23.0%)	4.16	NS
	Borderline-Clinical	28 (54.9%)	17 (33.3%)	6 (11.8%)		
**OPPOSITIONAL DEFIANT PROBLEMS**	Normal	203 (43.8%)	152 (32.8%)	109 (23.5%)	4.99	NS
	Borderline-Clinical	34 (46.6%)	30 (41.1%)	9 (12.3%)		
**CONDUCT PROBLEMS**	Normal	207 (43.7%)	155 (32.7%)	112 (23.6%)	6.92	**0.05**
	Borderline-Clinical	30 (47.6%)	27 (42.9%)	6 (9.5%)		
**SLUGGISH COGNITIVE PROBLEMS**	Normal	202 (43.0%)	160 (34.0%)	108 (23.0%)	2.90	NS
	Borderline-Clinical	35 (52.2%)	22 (32.8%)	10 (14.9%)		
**OBSESSIVE COMPULSIVE PROBLEMS**	Normal	204 (43.7%)	157 (33.6%)	106 (22.7%)	1.10	NS
	Borderline-Clinical	33 (47.1%)	25 (35.7%)	12 (10.2%)		
	Normal	195 (42.8%)	153 (33.6%)	108 (23.7%)	5.41	NS

**POST TRAUMATIC STRESS PROBLEMS**	Borderline-Clinical	42 (51.9%)	29 (35.8%)	10 (12.3%)

Supplemental Table S2. Normal and borderline-clinical YSR scores at initial assessment in subjects with obesity, overweight and normal BMI.

YSR Scales Initial Assessment	SCORE	OBESITY	OVERWEIGHT	NORMAL BMI	X^2	P-value
**ACTIVITIES**	Normal	60 (47.6%)	46 (36.5%)	20 (15.9%)	0.09	NS
	Borderline-Clinical	24 (47.1%)	18 (35.3%)	9 (17.6%)		
**SOCIAL**	Normal	68 (46.3%)	56 (38.1%)	23 (15.6%)	3.13	NS
	Borderline-Clinical	11 (45.8%)	6 (25.0%)	7 (29.2%)		
**TOTAL COMPETENCE**	Normal	45 (45.5%)	38 (38.4%)	16 (16.2%)	0.19	NS
	Borderline-Clinical	33 (48.5%)	24 (35.3%)	11 (16.2%)		
**ANXIOUS-DEPRESSED**	Normal	75 (48.1%)	56 (35.9%)	25 (16.0%)	0.91	NS
	Borderline-Clinical	7 (38.9%)	7 (38.9%)	4 (22.2%)		
**WITHDRAWN-DEPRESSED**	Normal	78 (48.1%)	58 (35.8%)	26 (16.0%)	1.44	NS
	Borderline-Clinical	4 (33.3%)	5 (41.7%)	3 (25.0%)		
**SOMATIC COMPLAINTS**	Normal	78 (47.9%)	58 (35.6%)	27 (16.6%)	0.78	NS
	Borderline-Clinical	4 (36.4%)	5 (45.5%)	2 (18.2%)		
**SOCIAL PROBLEMS**	Normal	78 (48.4%)	59 (36.6%)	24 (14.9%)	4.31	NS
	Borderline-Clinical	4 (30.8%)	4 (30.8%)	5 (38.5%)		
**THOUGHT PROBLEMS**	Normal	80 (47.1%)	62 (36.5%)	28 (16.5%)	0.77	NS
	Borderline-Clinical	2 (50.0%)	1 (25.0%)	1 (25.0%)		
**ATTENTION PROBLEMS**	Normal	78 (47.6%)	61 (37.2%)	25 (15.2%)	3.79	NS
	Borderline-Clinical	4 (40.0%)	2 (20.0%)	4 (40.0%)		
**RULE BREAKING BEHAVIOUR**	Normal	80 (47.3%)	60 (35.5%)	29 (17.2%)	1.23	NS
	Borderline-Clinical	2 (40.0%)	3 (60.0%)	-		
**AGGRESSIVE BEHAVIOUR**	Normal	76 (46.9%)	60 (37.0%)	26 (16.0%)	1.19	NS
	Borderline-Clinical	6 (50.0%)	3 (25.0%)	3 (25.0%)		
**INTERNALIZING PROBLEMS**	Normal	70 (50.0%)	47 (33.6%)	23 (16.4%)	2.71	NS

	Borderline-Clinical	12 (35.3%)	16 (47.1%)	6 (17.6%)		
**EXTERNALIZING PROBLEMS**	Normal	71 (47.3%)	56 (37.3%)	23 (15.3%)	1.62	NS
	Borderline-Clinical	11 (45.8%)	7 (29.2%)	6 (25.0%)		
**TOTAL PROBLEMS**	Normal	67 (47.5%)	51 (36.2%)	23 (16.3%)	0.17	NS
	Borderline-Clinical	15 (45.5%)	12 (36.4%)	6 (18.2%)		
**AFFECTIVE PROBLEMS**	Normal	75 (49.0%)	53 (34.6%)	25 (16.3%)	2.04	NS
	Borderline-Clinical	7 (33.3%)	10 (47.6%)	4 (19.0%)		
**ANXIETY PROBLEMS**	Normal	76 (46.9%)	60 (37.0%)	26 (16.0%)	1.19	NS
	Borderline-Clinical	6 (50.0%)	3 (25.0%)	3 (25.0%)		
**SOMATIC PROBLEMS**	Normal	78 (47.3%)	59 (35.8%)	28 (17.0%)	2.63	NS
	Borderline-Clinical	4 (44.4%)	4 (44.4%)	1 (11.1%)		
**ATTENTION DEFICIT-HYPERACTIVITY PROBLEMS**	Normal	80 (47.1%)	63 (37.1%)	27 (15.9%)	3.78	NS
	Borderline-Clinical	2 (50.0%)	-	2 (50.0%)		
**OPPOSITIONAL DEFIANT PROBLEMS**	Normal	79 (48.2%)	61 (37.2%)	24 (14.6%)	6.54	**0.05**
	Borderline-Clinical	3(30.0%)	2 (20.0%)	5 (50.0%)		
**CONDUCT PROBLEMS**	Normal	82 (47.1%)	63 (36.2%)	29 (16.7%)	-	-
	Borderline-Clinical	-	-	-		
**OBSESSIVE COMPULSIVE PROBLEMS**	Normal	77 (46.7%)	60 (36.4%)	28 (17.0%)	0.28	NS
	Borderline-Clinical	5 (55.6%)	3 (33.3%)	1 (11.1%)		
**POST TRAUMATIC STRESS PROBLEMS**	Normal	79 (49.1%)	57 (35.4%)	25 (15.5%)	4.02	NS
	Borderline-Clinical	3 (23.1%)	6 (46.2%)	4 (30.8%)		
**POSITIVE QUALITIES**	Normal	80 (47.3%)	61 (36.1%)	28 (16.6%)	0.51	NS
	Borderline-Clinical	2 (40.0%)	2 (40.0%)	1 (20.0%)		

Supplemental Table S3. Comparison of normal and borderline/clinical CBCL scores between initial and annual assessment in subjects with obesity (N=236), overweight (N=182), normal BMI (N=119), and all subjects (N=537).

CBCL Scales	Scores		Annual Assessment															
			OBESITY		X^2	P-value	OVERWEIGHT		X^2	P-value	NORMAL BMI		X^2	P-value	ALL SAMPLE		X^2	P-value
			Normal	Borderline-Clinical			Normal	Borderline-Clinical			Normal	Borderline-Clinical			Normal	Borderline-Clinical		
**ACTIVITIES**	**Initial Assessment**	Normal	118 (74.2%)	41 (25.8%)	6.52	**0.01**	101 (77.1%)	30 (22.9%)	21.43	**0.01**	70 (20%)	20 (22.2%)	12.28	**0.01**	289 (76.1%)	91 (23.9%)	35.71	**0.01**
		Borderline-Clinical	45 (57.7%)	33 (42.3%)			21 (41.2%)	30 (58.8%)			12 (42.9%)	16 (57.1%)			78 (49.7%)	79 (50.3%)		
**SOCIAL**		Normal	182 (94.3%)	11 (5.7%)	15.85	**0.01**	153 (95%)	8 (5%)	31.49	**0.01**	95 (93.1%)	7 (6.9%)	9.00	**0.01**	430 (94.3%)	26 (5.7%)	50.66	**0.01**
		Borderline-Clinical	33 (75%)	11 (25%)			12 (57.1%)	9 (42.9%)			11 (68.8%)	5 (31.3%)			56 (69.1%)	25 (30.9%)		
**SCHOOL**		Normal	220 (97.3%)	6 (2.7%)	29.49	**0.01**	175 (100%)	0 (0%)	50.55	**0.01**	111 (98.2%)	2 (1.8%)	40.01	**0.01**	506 (98.6%)	8 (1.6%)	101.38	**0.01**
		Borderline-Clinical	7 (63.6%)	4 (36.4%)			5 (71.4%)	2 (28.6%)			2 (40%)	3 (80%)			14 (60.9%)	9 (39.1%)		
**TOTAL COMPETENCE**		Normal	106 (75.7%)	34 (24.3%)	10.49	**0.01**	87 (71.3%)	35 (28.7%)	9.18	**0.01**	62 (78.5%)	17 (21.5%)	6.20	**0.05**	255 (74.8%)	86 (25.2%)	25.33	**0.01**
		Borderline-Clinical	54 (55.7%)	43 (44.3%)			29 (48.3%)	31 (51.7%)			22 (56.4%)	17 (43.6%)			105 (53.6%)	91 (46.4%)		
**ANXIOUS-DEPRESSED**		Normal	170 (91.9%)	15 (8.1%)	32.82	**0.01**	135 (88.8%)	17 (11.2%)	9.72	**0.01**	92 (92.9%)	7 (7.1%)	31.85	**0.01**	397 (91.1%)	39 (8.9%)	68.20	**0.01**
		Borderline-Clinical	31 (59.6%)	21 (40.4%)			20 (66.7%)	10 (33.3%)			8 (42.1%)	11 (57.9%)			59 (58.4%)	42 (41.6%)		

**WITHDRAWN-DEPRESSED**	Normal	159 (90.9%)	16 (9.1%)	42.14	**0.01**	138 (92%)	12 (8%)	13.41	**0.01**	92 (89.3%)	11 (10.7%)	5.73	**0.05**	389 (90.9%)	39 (9.1%)	64.94	**0.01**
	Borderline-Clinical	33 (53.2%)	29 (46.8%)			22 (66.8%)	10 (31.3%)			10 (66.7%)	5 (33.3%)			65 (59.6%)	44 (40.4%)		
**SOMATIC COMPLAINTS**	Normal	187 (92.1%)	16 (7.9%)	24.98	**0.01**	143 (93.5%)	10 (6.5%)	3.69	NS	95 (90.5%)	10 (9.5%)	2.16	NS	425 (92.2%)	36 (7.8%)	27.02	**0.01**
	Borderline-Clinical	21 (61.8%)	13 (38.2%)			24 (82.8%)	5 (17.2%)			10 (76.9%)	3 (23.1%)			55 (72.4%)	21 (27.6%)		
**SOCIAL PROBLEMS**	Normal	178 (92.7%)	14 (7.3%)	44.90	**0.01**	142 (94%)	9 (6%)	3.74	NS	99 (90%)	11 (10%)	0.05	NS	419 (92.5%)	34 (7.5%)	42.71	**0.01**
	Borderline-Clinical	24 (53.3%)	21 (46.7%)			26 (83.9%)	5 (16.1%)			7 (87.5%)	1 (12.5%)			57 (67.9%)	27 (32.1%)		
**THOUGHT PROBLEMS**	Normal	184 (92%)	16 (8%)	29.08	**0.01**	147 (95.5%)	7 (4.5%)	0.01	NS	95 (89.6%)	11 (10.4%)	0.43	NS	426 (92.6%)	34 (7.4%)	38.70	**0.01**
	Borderline-Clinical	22 (59.5%)	15 (40.5%)			21 (75%)	7 (25%)			10 (83.3%)	2 (16.7%)			53 (68.8%)	24 (31.2%)		
**ATTENTION PROBLEMS**	Normal	198 (94.3%)	12 (5.7%)	9.29	**0.01**	157 (94%)	10 (6%)	0.01	NS	102 (91.9%)	9 (8.1%)	0.32	NS	457 (93.6%)	31 (6.4%)	6.57	**0.01**
	Borderline-Clinical	21 (77.8%)	6 (22.2%)			14 (93.3%)	1 (6.7%)			6 (85.7%)	1 (14.3%)			41 (83.7%)	8 (16.3%)		
**RULE BREAKING BEHAVIOUR**	Normal	203 (94.4%)	12 (5.6%)	24.74	**0.01**	144 (92.9%)	11 (7.1%)	0.52	NS	104 (92.9%)	8 (7.1%)	0.46	NS	451 (93.6%)	31 (6.4%)	12.06	**0.01**
	Borderline-Clinical	14 (63.6%)	8 (36.4%)			24 (88.9%)	3 (11.1%)			6 (100%)	0 (0%)			44 (80%)	11 (20%)		
**AGGRESSIVE**	Normal	177 (91.2%)	17 (8.8%)	23.74	**0.01**	143 (94.1%)	9 (5.9%)	12.73	**0.01**	100 (91.7%)	9 (8.3%)	5.72	**0.01**	420 (92.3%)	35 (7.7%)	43.32	**0.01**

**BEHAVIOUR**	Borderline-Clinical	27 (62.8%)	16 (37.2%)			22 (73.3%)	8 (26.7%)			6 (66.7%)	3 (33.3%)			55 (67.1%)	27 (32.9%)		
**INTERNALIZING PROBLEMS**	Normal	121 (85.8%)	20 (14.2%)	35.80	**0.01**	98 (84.5%)	18 (15.5%)	7.78	**0.01**	81 (95.3%)	4 (4.7%)	49.43	**0.01**	300 (87.7%)	42 (12.3%)	78.80	**0.01**
	Borderline-Clinical	48 (50%)	48 (50%)			44 (66.7%)	22 (33.3%)			12 (36.4%)	21 (63.6%)			104 (53.3%)	91 (46.7%)		
**EXTERNALIZING PROBLEMS**	Normal	150 (89.8%)	17 (10.2%)	43.04	**0.01**	123 (92.5%)	10 (7.5%)	26.11	**0.01**	86 (85.1%)	15 (14.9%)	2.19	NS	359 (89.5%)	42 (10.5%)	69.36	**0.01**
	Borderline-Clinical	36 (51.4%)	34 (48.6%)			30 (61.2%)	19 (38.8%)			12 (70.6%)	5 (29.4%)			78 (57.4%)	58 (42.6%)		
**TOTAL PROBLEMS**	Normal	126 (85.1%)	22 (14.9%)	38.77	**0.01**	113 (89.7%)	13 (10.3%)	14.91	**0.01**	87 (91.6%)	8 (8.4%)	17.60	**0.01**	326 (88.3%)	43 (11.7%)	75.48	**0.01**
	Borderline-Clinical	42 (47.2%)	47 (52.8%)			37 (66.1%)	19 (33.9%)			13 (56.5%)	10 (43.5%)			92 (54.8%)	76 (45.2%)		
**AFFECTIVE PROBLEMS**	Normal	149 (88.7%)	19 (11.3%)	35.61	**0.01**	129 (90.2%)	14 (9.8%)	11.01	NS	88 (94.6%)	5 (5.4%)	17.67	**0.01**	366 (90.6%)	38 (9.4%)	65.89	**0.01**
	Borderline-Clinical	37 (53.6%)	32 (46.4%)			27 (69.2%)	12 (30.8%)			16 (64%)	9 (36%)			80 (60.2%)	53 (39.8%)		
**ANXIETY PROBLEMS**	Normal	154 (87.5%)	22 (12.5%)	18.07	**0.01**	119 (88.1%)	16 (11.9%)	3.68	NS	96 (93.2%)	7 (6.8%)	19.90	**0.01**	369 (89.1%)	45 (10.9%)	35.57	**0.01**
	Borderline-Clinical	38 (62.3%)	23 (37.7%)			36 (76.6%)	11 (23.4%)			8 (53.3%)	7 (46.7%)			82 (66.7%)	41 (33.3%)		
**SOMATIC PROBLEMS**	Normal	187 (92.6%)	15 (7.4%)	25.28	**0.01**	145 (94.8%)	8 (5.2%)	2.09	NS	95 (89.6%)	11 (10.2%)	0.43	NS	427 (92.6%)	34 (7.4%)	22.78	**0.01**

	Borderline-Clinical	22 (62.9%)	13 (37.1%)			25 (86.2%)	4 (13.8%)			10 (83.2%)	2 (16.7%)			57 (75%)	19 (25%)		
**ATTENTION DEFICIT-HYPERACTIVITY PROBLEMS**	Normal	198 (94.7%)	11 (5.3%)	28.35	**0.01**	155 (93.9%)	10 (6.1%)	3.1	NS	95 (89.6%)	11 (10.4%)	0.43	NS	455 (93.6%)	31 (6.4%)	26.69	**0.01**
	Borderline-Clinical	18 (64.3%)	10 (35.7%)			14 (82.4%)	3 (17.6%)			10 (83.3%)	2 (16.7%)			37 (72.5%)	14 (27.5%)		
**OPPOSITIONAL DEFIANT PROBLEMS**	Normal	191 (94.1%)	12 (5.9%)	19.09	**0.01**	142 (93.4%)	10 (6.6%)	8.3	NS	102 (91.1%)	10 (8.9%)	0.40	NS	431 (92.9%)	33 (7.1%)	25.80	**0.01**
	Borderline-Clinical	24 (70.6%)	10 (29.4%)			23 (76.7%)	7 (23.3%)			5 (83.3%)	1 (16.7%)			54 (74%)	19 (26%)		
**CONDUCT PROBLEMS**	Normal	191 (92.3%)	16 (7.7%)	21.73	**0.01**	146 (94.2%)	9 (5.8%)	2.81	NS	98 (89.9%)	11 (10.1%)	1.24	NS	440 (92.8%)	34 (7.2%)	18.56	**0.01**
	Borderline-Clinical	19 (63.3%)	11 (36.7%)			23 (85.2%)	4 (14.8%)			7 (77.8%)	2 (22.2%)			48 (76.2%)	15 (23.8%)		
**SLUGGISH COGNITIVE PROBLEMS**	Normal	183 (90.6%)	19 (9.4%)	16.24	**0.01**	150 (93.8%)	10 (6.3%)	1.59	NS	70 (77.8%)	20 (22.2%)	12.28	**0.01**	431 (91.7%)	39 (8.3%)	24.49	**0.01**
	Borderline-Clinical	23 (65.7%)	12 (34.3%)			19 (86.4%)	3 (13.6%)			12 (42.9%)	16 (57.1%)			48 (71.6%)	19 (28.4%)		
**OBSESSIVE COMPULSIVE PROBLEMS**	Normal	194 (95.1%)	10 (4.9%)	49.50	**0.01**	148 (94.3%)	9 (5.7%)	3.42	NS	95 (93.1%)	7 (6.9%)	9.00	**0.01**	439 (94%)	28 (6%)	51.10	**0.01**
	Borderline-Clinical	18 (54.5%)	15 (45.5%)			21 (84%)	4 (16%)			11 (68.8%)	5 (31.3%)			47 (67.1%)	23 (32.9%)		
**POST TRAUMATIC STRESS PROBLEMS**	Normal	178 (91.3%)	17 (8.7%)	21.56	**0.01**	144 (94.1%)	9 (5.9%)	7.06	**0.01**	111 (98.2%)	2 (1.8%)	40.01	**0.01**	423 (92.8%)	33 (7.2%)	47.19	**0.01**
	Borderline-Clinical	27 (64.3%)	15 (13.5%)			23 (79.3%)	6 (20.7%)			2 (40%)	3 (60%)			54 (66.7)	27 (33.3%)		

Supplemental Table S4. Comparison of normal and borderline/clinical YSR scores between initial and annual assessment in subjects with obesity (N=80), overweight (N=61), normal BMI (N=28), and all subjects (N=169).

			**Annual Assessment**															
			**OBESITY**				**OVERWEIGHT**				**NORMAL BMI**				**ALL SAMPLE**			
**YSR Scales**	**Scores**		Normal	Borderline-Clinical	**X^2**	**P-value**	Normal	Borderline-Clinical	**X^2**	**P-value**	Normal	Borderline-Clinical	**X^2**	**P-value**	Normal	Borderline-Clinical	**X^2**	**P-value**
**ACTIVITIES**	**Initial Assessment**	Normal	39 (65%)	21 (35%)	11.02	**0.01**	35 (76.1%)	11 (23.9%)	10.27	**0.01**	16 (80%)	4 (20%)	1.87	NS	90 (71.4%)	36 (28.6%)	22.03	**0.01**
		Borderline-Clinical	6 (25%)	18 (75%)			6 (33.3%)	12 (66.7%)			5 (55.6%)	4 (44.4%)			17 (33.3%)	34 (66.7%)		
**SOCIAL**		Normal	60 (88.2%)	8 (11.8%)	4.44	**0.05**	47 (83.9%)	9 (16.1%)	0.01	NS	20 (87%)	3 (13%)	0.93	NS	127 (86.4%)	20 (13.6%)		**0.05**
		Borderline-Clinical	7 (63.6%)	4 (36.4%)			5 (83.3%)	1 (16.7%)			5 (71.4%)	2 (28.6%)			17 (%)	7 (%)		
**TOTAL COMPETENCE**		Normal	28 (62.2%)	17 (37.8%)	5.09	**0.05**	26 (68.4%)	12 (31.6%)	11.10	**0.01**	11 (68.8%)	5 (31.3%)	4.49	**0.05**	65 (65.7%)	34 (34.3%)	19.51	**0.01**
		Borderline-Clinical	12 (36.4%)	21 (63.6%)			6 (25%)	18 (75%)			3 (27.3%)	8 (72.7%)			21 (30.9%)	47 (69.1%)		
**ANXIOUS-DEPRESSED**		Normal	67 (98.5%)	1 (1.5%)	21.53	**0.01**	51 (98.1%)	1 (1.9%)	9.07	**0.01**	22 (95.7%)	1 (4.3%)	18.65	**0.01**	140 (97.9%)	3 (2.1%)	47.97	**0.01**
		Borderline-Clinical	4 (57.1%)	3 (42.9%)			5 (71.4%)	2 (28.6%)			0 (0%)	3 (100%)			9 (52.9%)	8 (47.1%)		
**WITHDRAWN-DEPRESSED**		Normal	70 (98.6%)	1 (1.4%)	8.12	**0.01**	51 (94.4%)	3 (5.6%)	24.25	**0.01**	23 (95.8%)	1 (4.2%)	16.61	**0.01**	144 (96.6%)	5 (3.4%)	53.65	**0.01**
		Borderline-Clinical	3 (75%)	1 (25%)			1 (20%)	4 (80%)			0 (0%)	2 (100%)			4 (36.4%)	7 (63.6%)		

**SOMATIC COMPLAINTS**	Normal	72 (%)	0 (0%)	-	NS	51 (94.4%)	3 (5.6%)	1.51	NS	23 (92%)	2 (8%)	0.08	NS	146 (96.7%)	5 (3.3%)	1.43	NS
	Borderline-Clinical	3 (%)	0 (0%)			4 (80%)	1 (20%)			1 (100%)	0 (0%)			8 (88.9%)	1 (11.1%)		
**SOCIAL PROBLEMS**	Normal	69 (97.2%)	2 (2.8%)	0.11	NS	53 (96.4%)	2 (3.6%)	3.52	NS	21 (95.5%)	1 (4.5%)	0.18	NS	143 (96.6%)	5 (3.4%)	0.75	NS
	Borderline-Clinical	4 (100%)	0 (0%)			3 (75%)	1 (25%)			4 (100%)	0 (0%)			11 (91.7%)	1 (8.3%)		
**ATTENTION PROBLEMS**	Normal	70 (97.2%)	2 (2.8%)	23.28	**0.01**	55 (96.5%)	2 (3.5%)	0.07	NS	22 (95.7%)	1 (4.3%)	3.14	NS	147 (96.7%)	5 (3.3%)	18.72	**0.01**
	Borderline-Clinical	1 (33.3%)	2 (66.7%)			2 (100%)	0 (0%)			2 (66.7%)	1 (33.3%)			5 (62.7%)	3 (37.5%)		
**RULE BREAKING BEHAVIOUR**	Normal	72 (98.6%)	1 (1.4%)	17.73	**0.01**	55 (98.2%)	1 (1.8%)	24.83	**0.01**	26 (100%)	0 (0%)	-	NS	153 (98.7%)	2 (1.3%)	55.14	**0.01**
	Borderline-Clinical	1 (50%)	1 (50%)			1 (33.3%)	2 (66.7%)			0 (0%)	0 (0%)			2 (40%)	3 (60%)		
**AGGRESSIVE BEHAVIOUR**	Normal	67 (97.1%)	2 (2.9%)	2.72	NS	56 (98.2%)	1 (1.8%)	38.64	**0.01**	23 (100%)	0 (0%)	16.61	**0.01**	146 (98%)	3 (2%)	40.69	**0.01**
	Borderline-Clinical	5 (83.3%)	1 (16.7%)			0 (0%)	2 (100%)			1 (33.3%)	2 (66.7%)			6 (54.5%)	5 (45.5%)		
**INTERNALIZING PROBLEMS**	Normal	61 (96.8%)	2 (3.2%)	12.45	NS	41 (95.3%)	2 (4.7%)	24.08	**0.01**	18 (85.7%)	3 (14.3%)	8.86	**0.01**	120 (94.5%)	7 (5.5%)	47.77	**0.01**
	Borderline-Clinical	8 (66.7%)	4 (33.3%)			6 (37.5%)	10 (62.5%)			1 (20%)	4 (80%)			15 (45.5%)	18 (54.5%)		
**EXTERNALIZING PROBLEMS**	Normal	63 (96.9%)	2 (3.1%)	22.55	**0.01**	50 (94.3%)	3 (5.7%)	19.18	**0.01**	21 (100%)	0 (0%)	14.24	**0.01**	134 (96.4%)	5 (3.6%)	55.08	**0.01**

	Borderline-Clinical	5 (50%)	5 (50%)			2 (33.3%)	4 (66.7%)			2 (40%)	3 (60%)			9 (42.9%)	12 (57.1%)		
**TOTAL PROBLEMS**	Normal	62 (100%)	0 (0%)	31.10	**0.01**	45 (95.7%)	2 (4.3%)	17.06	**0.01**	20 (95.2%)	1 (4.8%)	14.71	**0.01**	127 (97.7.%)	3 (2.3%)	60.64	**0.01**
	Borderline-Clinical	7 (53.8%)	6 (46.2%)			6 (50%)	6 (50%)			1 (20%)	4 (80%)			14 (46.7%)	16 (53.3%)		
**AFFECTIVE PROBLEMS**	Normal	67 (98.5%)	1 (1.5%)	12.13	**0.01**	47 (95.9%)	2 (4.1%)	11.72	**0.01**	22 (95.7%)	1 (4.3%)	18.65	**0.01**	136 (97.1%)	4 (2.9%)	41.63	**0.01**
	Borderline-Clinical	5 (71.4%)	2 (28.6%)			6 (60%)	4 (40%)			0 (0%)	3 (100%)			11 (55%)	9 (45%)		
**ANXIETY PROBLEMS**	Normal	67 (97.1%)	2 (2.9%)	2.72	NS	56 (100%)	0 (0%)	18.98	**0.01**	22 (95.7%)	1 (4.3%)	3.14	NS	145 (98%)	3 (2%)	16.23	**0.01**
	Borderline-Clinical	5 (83.3%)	1 (16.7%)			2 (66.7%)	1 (33.3%)			2 (66.7%)	1 (33.3%)			9 (75%)	3 (25%)		
**SOMATIC PROBLEMS**	Normal	71 (98.6%)	1 (1.4%)	11.32	**0.01**	52 (94.5%)	3 (5.5%)	9.53	**0.01**	24 (96%)	1 (4%)	0.04	NS	147 (96.7%)	5 (3.3%)	18.72	**0.01**
	Borderline-Clinical	2 (66.7%)	1 (33.3%)			2 (50%)	2 (50%)			1 (100%)	0 (0%)			5 (62.5%)	3 (37.5%)		
**ATTENTION DEFICIT-HYPERACTIVITY PROBLEMS**	Normal	74 (100%)	0 (0%)	75	**0.01**	57 (96.6%)	2 (3.4%)	-	NS	23 (95.8%)	1 (4.2%)	5.46	**0.05**	154 (98.1%)	3 (1.9%)	40.77	**0.01**
	Borderline-Clinical	0 (0%)	1 (100%)			0 0%)	0 (0%)			1 (50%)	1 (50%)			1 (33.3%)	2 (66.7%)		
**OPPOSITIONAL DEFIANT PROBLEMS**	Normal	68 (94.4%)	4 (5.6%)	3.57	NS	51 (89.5%)	6 (10.5%)	2.87	NS	21 (95.5%)	1 (4.5%)	12.90	0.01	140 (92.7%)	11 (7.3%)	21.99	**0.01**
	Borderline-Clinical	2 (66.7%)	1 (33.3%)			1 (50%)	1 (50%)			1 (25%)	3 (75%)			4 (44.4%)	5 (55.6%)		

**CONDUCT PROBLEMS**	Normal	72 (96%)	3 (4%)	-	NS	56 (94.9%)	3 (5.1%)	-	NS	26 (100%)	0 (0%)	-	NS	154 (96.3%)	6 (3.8%)	-	NS
	Borderline-Clinical	0 (0%)	0 (0%)			0 (0%)	0 (0%)			0 (0%)	0 (0%)			0 (0%)	0 (0%)		
**OBSESSIVE COMPULSIVE PROBLEMS**	Normal	69 (98.6%)	1 (1.4%)	18.08	**0.01**	51 (91.1%)	5 (8.9%)	0.29	NS	24 (96%)	1 (4%)	12.48	**0.01**	144 (95.4%)	7 (4.6%)	11.93	**0.01**
	Borderline-Clinical	3 (60%)	2 (40%)			3 (100%)	0 (0%)			0 (0%)	1 (100%)			6 (66.7%)	3 (33.3%)		
**POST TRAUMATIC STRESS PROBLEMS**	Normal	72 (100%)	0 (0%)	49.31	**0.01**	48 (90.6%)	5 (9.4%)	0.61	NS	21 (91.3%)	2 (8.7%)	6.85	**0.05**	141 (95.3%)	7 (4.7%)	14.18	**0.01**
	Borderline-Clinical	1 (33.3%)	2 (66.7%)			6 (100%)	0 (0%)			1 (33.3%)	2 (66.7%)			6 (66.7%)	4 (33.3%)		
**POSITIVE QUALITIES**	Normal	71 (97.3%)	2 (2.7%)	0.05	NS	56 (98.2%)	1 (1.5%)	13.73	NS	25 (100%)	0 (0%)	26.00	**0.01**	152 (98.1%)	3 (1.9%)	23.18	**0.01**
	Borderline-Clinical	2 (100%)	0 (0%)			1 (50%)	1 (50%)			0 (0%)	1 (100%)			3 (60%)	2 (40%)		

Supplemental Table S5. Psychometric scores categorized by gender in CBCL in subjects with obesity (N=236), overweight (N=182), normal BMI (N=119), and all subjects (N=537) at initial and annual assessment.

CBCL	Initial Evaluation									Annual Evaluation									P between time points	
	MALE				FEMALE				P between Gender	MALE				FEMALE				P between Gender		
	Obesity	Overweight	Normal BMI	P within baseline	Obesity	Overweight	Normal BMI	P within baseline		Obesity	Overweight	Normal BMI	P within Follow up	Obesity	Overweight	Normal BMI	P within Follow up		MALE	FEMALE
**ACTIVITIES**	20.41 ± 22.07	23.62 ± 20.98	21.10 ± 23.90	NS	26.81 ± 23.26	26.38 ± 25.34	28.64 ± 26.86	NS	**0.01**	21.78 ± 22.57	24.12 ± 21.82	18.76 ± 20.04	NS	26.07 ± 27.51	22.80 ± 20.64	27.64 ± 23.76	NS	**0.05**	NS/NS /NS	NS/ NS/ NS
**SOCIAL**	34.95± 25.48	38.10 ± 22.99	45.10 ± 27.83#	**0.05**	31.53 ± 26.56	38.24 ± 23.59	40.21± 26.44#	**0.05**	NS	37.86 ± 25.91	41.59 ± 26.66	40.77 ± 22.30	NS	38.17 ± 23.44	39.40 ± 24.33	40.34 ± 24.26	NS	NS	**0.05/** NS/NS	**0.05/**NS /NS
**SCHOOL**	50.55± 22.60	56.96 ± 18.67#	48.16 ± 26.33	**0.05**	51.37 ± 20.82	54.48 ± 20.63	56.77 ± 16.57	NS	NS	53.09 ± 21.64	55.38 ± 16.96	57.72 ± 19.80	NS	55.27 ± 20.13	56.31 ± 17.31	60.25 ± 15.48	NS	NS	**0.05/** NS/**0.05**	NS/NS /NS
**TOTAL COMPETENCE**	26.69 ± 24.32	33.74 ± 25.78	34.29 ± 29.92	NS	26.60 ± 24.65	31.02 ± 24.15	33.79 ± 26.99	NS	NS	29.83 ± 24.02	34.48 ± 25.80	32.65 ± 23.24	NS	28.45 ± 26.64	30.64 ± 24.87	34.33 ± 26.34	NS	NS	NS/NS /NS	NS/ NS/NS
**ANXIOUS-DEPRESSED**	72.01 ± 17.93	71.46 ± 18.70	66.65 ± 15.77	NS	70.49 ± 17.68	66.10 ± 16.46	65.82 ± 17.19	NS	**0.05**	68.48 ± 18.45	67.20 ± 17.29	66.54 ± 14.31	NS	63.57 ± 15.89	65.42 ± 15.96	63.66 ± 17.13	NS	NS	**0.01/0.01**/NS	**0.01/**NS /NS
**WITHDRAWN-DEPRESSED**	73.48 ±	70.88 ± 17.53	64.68 ± 15.76#	**0.01**	68.42 ± 18.09	64.04 ± 16.80	64.81± 16.54	NS	**0.01**	71.72 ± 19.52	66.07 ± 16.10#	63.79 ± 15.22#	**0.05**	71.24 ± 17.12	65.19 ± 16.24	62.65 ± 16.23#	**0.01**	NS	**0.01/0.05** /NS	NS/ NS/NS

	18.69																			
**SOMATIC COMPLAINTS**	68.65 ± 16.37	68.00 ± 17.22	61.48 ± 16.14[#]	**0.05**	71.00 ± 17.74	68.57 ± 17.49	67.37 ± 16.27	NS	NS	68.28 ± 18.13	64.94 ± 15.13	58.67 ± 13.72[#+]	**0.05**	63.57 ± 15.33	67.17 ± 16.26	64.97 ± 16.09	NS	NS	**0.01**/ **0.01**/NS	**0.01/0.05 /0.05**
**SOCIAL PROBLEMS**	72.5 ± 16.71	71.11 ± 16.31	65.00 ± 15.52[#]	**0.05**	71.99 ± 16.85	65.02 ± 15.25[#]	65.04 ± 14.78[#]	**0.01**	**0.01**	68.86 ± 18.22	65.23 ± 15.40	63.18 ± 13.08	NS	68.90 ± 16.30	64.75 ± 15.28	62.09 ± 15.33[#]	**0.05**	NS	**0.01/0.01** /NS	**0.05**/NS /**0.05**
**THOUGHT PROBLEMS**	66.30 ± 17.45	64.89 ± 16.57	61.65 ± 16.29	NS	63.18 ± 16.21	63.21 ± 16.87	61.45 ± 14.79	NS	NS	66.04 ± 17.63	62.12 ± 15.67	61.21 ± 14.23	NS	62.21 ± 18.25	60.82 ± 15.28	61.94 ± 15.79	NS	NS	NS/ **0.05**/NS	NS/**0.05** /NS
**ATTENTION PROBLEMS**	64.93 ± 15.41	63.64 ± 14.55	61.58 ± 12.36	NS	62.83 ± 13.41	60.51 ± 11.91	61.99 ± 13.77	NS	NS	64.69 ± 15.79	60.89 ± 13.91	62.21 ± 13.39	NS	63.12 ± 14.18	58.79 ± 11.25	61.06 ± 13.59	NS	NS	0.**05/0.05** /NS	NS/NS/NS
**RULE BREAKING BEHAVIOUR**	65.49 ± 16.54	64.46 ± 16.67	66.97 ± 15.80	NS	64.58 ± 14.79	64.38 ± 15.01	63.54 ± 13.80	NS	NS	65.14 ± 16.09	63.25 ± 14.77	59.95 ± 13.26	NS	61.29 ± 12.96	61.60 ± 14.63	60.30 ± 12.55	NS	NS	**0.01/0.05 /0.05**	NS/ **0.01/ 0.01**
**AGGRESSIVE BEHAVIOUR**	67.39 ± 18.45	66.90 ± 17.98	64.03 ± 16.58	NS	66.61 ± 17.12	64.33 ± 16.88	61.05 ± 13.12[#]	**0.05**	NS	65.11 ± 17.51	65.42 ± 17.46	64.00 ± 13.40	NS	65.00 ± 17.90	62.61 ± 14.45	60.26 ± 14.92	NS	NS	**0.05/0.05**/NS	NS/ NS/NS
**INTERNALIZING PROBLEMS**	66.04 ± 29.83	63.71 ± 29.31	56.94 ± 27.96	NS	63.93 ± 29.01	54.39 ± 31.38[#]	55.19 ± 29.16	**0.05**	**0.05**	60.25 ± 33.22	55.72 ± 30.46	54.56 ± 24.50	NS	53.05 ± 31.00	55.09 ± 28.16	51.00 ± 28.00	NS	NS	**0.01**/ **0.01**/ NS	**0.01**/ NS/ NS
**EXTERNALIZING PROBLEMS**	55.42 ±	54.25 ± 30.26	50.65 ± 32.44	NS	55.97 ± 28.18	52.54 ± 28.45	49.45 ± 26.43	NS	NS	50.86 ± 32.10	51.32 ± 30.71	51.03 ± 24.29	NS	50.74 ± 30.15	50.05 ± 26.01	43.21 ± 28.96	NS	NS	**0.01**/ **0.05**/ NS	NS/ **0.05**/NS

	30.94																			
**TOTAL PROBLEMS**	62.47± 30.59	59.29 ± 30.28	51.23 ± 28.76	NS	62.28 ± 28.24	53.80 ± 30.39#	50.29 ± 28.23#	**0.05**	NS	55.59 ± 33.84	50.99 ± 30.64	49.08 ± 22.81	NS	53.90 ± 30.64	51.19 ± 27.94	43.56 ± 29.54	NS	NS	**0.01/0.01**/ NS	0.**01/0.05/0.05**
**AFFECTIVE PROBLEMS**	75.29 ± 18.59	72.38 ± 18.34	69.94 ± 19.22	NS	72.50 ± 18.58	68.66 ± 18.13	67.23 ± 17.97	NS	**0.05**	72.35 ± 19.64	65.85 ± 16.28#	71.31 ± 15.39	**0.05**	71.74 ± 18.02	71.05 ± 17.08	64.51 ± 17.08#+	**0.05**	NS	**0.01/ 0.05**/NS	NS/ NS/NS
**ANXIETY PROBLEMS**	73.92 ± 17.67	72.06 ± 19.33	65.58 ± 14.61#	**0.05**	71.63 ± 18.71	67.24 ± 17.79	67.37 ± 17.40	NS	**0.05**	69.28 ± 19.08	67.96 ± 18.01	66.44 ± 15.78	NS	67.05 ± 18.13	65.35 ± 16.77	65.01 ± 17.98	NS	NS	**0.01/0.05**/ NS	**0.01**/NS/ NS
**SOMATIC PROBLEMS**	60.90 ± 17.62	65.33 ± 18.18	66.07 ± 15.55	NS	67.92 ± 18.94	66.24 ± 18.12	66.42 ± 17.70	NS	NS	66.80 ± 18.27	63.04 ± 16.60	58.74± 13.60#	0.05	61.00 ± 15.60	67.12 ± 17.44	64.49 ± 17.53	NS	NS	NS/**0.01**/NS	NS/NS/ NS
**ATTENTION DEFICIT-HYPERACTIVITY PROBLEMS**	64.96 ± 17.03	62.83 ± 15.26	62.48 ± 13.66	NS	62.42 ± 14.25	60.64 ± 13.67	60.13 ± 13.61	NS	**0.05**	64.07 ± 16.56	60.94 ± 14.87	61.87± 15.52	NS	63.24 ± 14.83	58.53 ± 12.43	59.25 ± 13.04	NS	NS	**0.05/ 0.05**/NS	NS/NS / NS
**OPPOSITIONAL DEFIANT PROBLEMS**	66.20 ± 16.75	66.29 ± 15.76	65.290 ± 16.12	NS	65.35 ± 15.37	63.92 ± 16.01	60.99 ± 12.58	NS	NS	62.85 ± 15.04	65.86 ± 16.22	64.28 ± 12.43	NS	65.86 ± 16.21	62.29 ± 13.53	60.94 ± 13.71	NS	NS	**0.01**/ /NS /NS	NS/NS/NS
**CONDUCT PROBLEMS**	65.25 ± 16.53	64.80 ± 17.61	64.84 ± 15.73	NS	62.67 ± 16.09	63.00 ± 15.80	60.37 ± 12.82	NS	**0.05**	65.86 ± 17.41	63.27 ± 15.19	60.05 ± 12.13	NS	60.12 ± 13.73	59.99 ± 14.72	58.31 ± 13.03	NS	**0.05**	**0.05/0.05/0.05**	NS/ **0.01**/ NS

**SLUGGISH COGNITIVE PROBLEMS**	66.04 ± 17.92	63.74 ± 17.25	57.42 ± 14.48#	**0.05**	64.51 ± 16.56	60.87 ± 15.08	60.98 ± 15.25	NS	NS	63.86 ± 17.88	60.43 ± 15.47	59.00 ± 14.84	NS	63.57 ± 17.19	61.57 ± 16.19	61.56 ± 15.65	NS	NS	**0.05**/NS/NS	NS/NS/ NS
**OBSESSIVE COMPULSIVE PROBLEMS**	67.68 ± 17.42	68.34 ± 17.54	68.29 ± 16.44	NS	65.37 ± 15.51	63.29 ± 15.75	64.84 ± 15.50	NS	**0.05**	64.90 ± 17.33	65.19 ± 15.42	65.13 ± 15.03	NS	63.83 ± 16.64	63.23 ± 13.80	63.84 ± 14.84	NS	NS	**0.05/0.01/**NS	NS/NS/NS
**POST TRAUMATIC STRESS PROBLEMS**	70.24 ± 19.27	69.34 ± 18.58	62.77 ± 14.80#	**0.05**	67.03 ± 16.92	63.60 ± 16.50	62.90 ± 15.51	NS	**0.01**	68.42 ± 19.27	64.56 ± 17.37	64.10 ± 12.77	NS	66.98 ± 17.94	62.39 ± 16.33	61.83 ± 16.53	NS	NS	**0.01/0.05/**NS	NS/NS/NS

* All results are presented as mean ± SD. Subjects were classified as obese, overweight, or with normal BMI according to IOTF criteria at initial assessment. Tables present comparisons among three groups at both initial and annual assessment. All measured variables were compared by employing repeated-measures ANOVA. Significant main effects were revealed by the LSD posthoc test. Statistical significance was set at ($p < 0.05$, rounded to 0.05 in Table), while strong significance ($p < 0.01$, rounded to 0.01 in Table) is also noted. NS: nonsignificant ($p > 0.05$) difference. +: Indicates significant difference from Overweight group, #: Indicates significant difference from Obese group. p-values between two timepoints refer to obese, overweight, and normal BMI respectively.

Supplemental Table S6. Psychometric scores categorized by gender in YSR in subjects with obesity (N=80), overweight (N=61), normal BMI (N=28), and all subjects (N=169) at initial and annual assessment.

YSR	Initial Evaluation									Annual Evaluation									P between timepoints	
	MALE				FEMALE					MALE				FEMALE						
	Obesity	Overweight	Normal BMI	P within baseline	Obesity	Overweight	Normal BMI	P within baseline	P between Gender	Obesity	Overweight	Normal BMI	P within Follow up	Obesity	Overweight	Normal BMI	P within Follow up	P between Gender	MALE	FEMALE
**ACTIVITIES**	21.94 ± 22.35	20.90 ± 18.98	25.50 ± 30.38	NS	27.42 ± 26.17	32.36 ± 30.38	27.53 ± 25.05	NS	NS	18.54 ± 28.31	20.86 ± 22.10	17.43 ± 24.79	NS	18.93 ± 16.18	22.20 ± 21.67	33.64 ± 25.35$^{\#}$	**0.05**	NS	NS/NS / NS	NS/NS /NS
**SOCIAL**	37.00 ± 26.83	45.29 ± 24.88	37.50 ± 29.68	NS	35.00 ± 26.10	43.74 ± 24.54	40.62 ± 27.11	NS	NS	35.60 ± 28.71	55.43 ± 26.75$^{\#}$	43.10 ± 25.32	**0.01**	36.80 ± 20.86	43.48 ± 19.69	45.14 ± 27.54	NS	NS	NS/ NS/NS	NS/ NS/NS
**TOTAL COMPETENCE**	28.93 ± 25.02	31.39 ± 21.89	27.63 ± 17.35	NS	27.41 ± 26.83	33.17 ± 26.68	32.78 ± 25.82	NS	NS	24.72 ± 26.65	36.82 ± 23.90	26.00 ± 25.65	NS	20.93 ± 15.92	26.96 ± 24.09	39.32 ± 26.78$^{\#}$	**0.05**	NS	NS/NS /NS	NS/NS /NS
**ANXIOUS-DEPRESSED**	66.53 ± 17.12	66.94 ± 17.38	58.63 ± 11.52	NS	67.87 ± 16.17	65.81 ± 17.75	68.95 ± 19.51	NS	NS	61.52 ± 14.85	62.80 ± 13.77	67.92 ± 17.19	NS	64.00 ± 17.89	66.68 ± 16.07	61.45 ± 17.82	NS	NS	NS/**0.05**/NS	NS/NS /NS
**WITHDRAWN-DEPRESSED**	60.80 ± 14.50	61.84 ± 14.96	61.13 ± 16.86	NS	64.87 ± 15.33	66.09 ± 17.87	63.86 ± 17.58	NS	NS	59.56 ± 12.77	61.96 ± 16.53	61.42 ± 15.32	NS	73.13 ± 17.95	61.48 ± 15.07$^{\#}$	62.45 ± 18.90	**0.05**	NS	NS/NS /NS	NS/NS /NS
**SOMATIC COMPLAINTS**	66.02 ± 15.97	64.68 ± 14.77	60.13 ± 13.30	NS	59.55 ± 12.72	62.34 ± 16.49	59.29 ± 15.65	NS	NS	58.40 ± 11.381	60.52 ± 11.01	61.00 ± 10.55	NS	54.60 ± 5.88	59.16 ± 13.27	60.00 ± 18.00	NS	NS	**0.01/0.05**/NS	NS/NS /NS
**SOCIAL PROBLEMS**	64.51	61.00 ±	62.00 ± 13.28	NS	69.52 ± 16.45	64.19 ±	65.71 ± 19.49	NS	NS	54.92 ± 9.25	62.16 ± 14.41$^{\#}$	61.92 ± 14.25	**0.05**	61.27 ± 16.65	57.36 ± 8.31	55.85 ± 10.54	NS	NS	**0.05**/NS/NS	**0.01**/NS /NS

	± 14.37	15.01				16.36														
**THOUGHT PROBLEMS**	62.43 ± 14.70	61.29 ± 14.09	59.50 ± 9.91	NS	56.97 ± 9.68	61.41 ± 13.58	61.33 ± 13.03	NS	NS	58.88 ± 13.02	59.92 ± 13.19	57.17 ± 11.94	NS	58.88 ± 10.54	59.92 ± 10.00	57.17 ± 12.41	NS	NS	**0.05**/NS/NS	NS/NS /NS
**ATTENTION PROBLEMS**	59.24 ± 12.87	60.58 ± 15.17	60.38 ± 9.02	NS	64.16 ± 13.49	61.69 ± 12.00	65.95 ± 19.22	NS	NS	56.08 ± 12.28	60.16 ± 13.44	60.75 ± 13.19	NS	56.08 ± 16.71	60.16 ± 11.82	60.75 ± 8.20	NS	NS	NS/NS/NS	NS/NS /NS
**RULE BREAKING BEHAVIOUR**	57.18 ± 11.91	56.97 ± 12.96	56.38 ± 8.51	NS	60.84 ± 13.53	61.50 ± 15.52	57.38 ± 11.66	NS	NS	56.322 ± 9.25	58.40 ± 13.34	54.00 ± 5.11	NS	56.32 ± 7.89	58.40 ± 14.42	54.00 ± 6.40	NS	NS	NS/NS/NS	NS/NS /NS
**AGGRESSIVE BEHAVIOUR**	63.69 ± 15.91	61.06 ± 14.27	57.75 ± 7.86	NS	67.94 ± 15.17	62.06 ± 14.67	65.48 ± 19.14	NS	NS	56.52 ± 11.10	64.16 ± 17.82	63.83 ± 14.02	NS	56.52 ± 14.09	64.16 ± 13.83	63.83 ± 11.87	NS	NS	**0.05/NS/ 0.05**	**0.05/** NS/NS
**INTERNALIZING PROBLEMS**	53.12 ± 27.97	51.55 ± 31.00	40.13 ± 26.43	NS	55.74 ± 25.51	49.63 ± 33.31	48.86 ± 34.80	NS	NS	42.28 ± 26.71	47.28 ± 27.27	49.00 ± 32.43	NS	42.28 ± 30.36	47.24 ± 26.33	49.00 ± 35.88	NS	NS	NS/ **0.05**/NS	NS/NS /NS
**EXTERNALIZING PROBLEMS**	45.51 ± 27.23	44.29 ± 25.34	44.25 ± 17.31	NS	55.61 ± 25.30	46.16 ± 29.94	45.57 ± 32.40	NS	NS	36.88 ± 24.07	48.44 ± 28.29	49.42 ± 18.57	NS	36.88 ± 26.73	48.44 ± 26.35	49.42 ± 23.99^{+}	**0.01**	NS	NS/NS/**0.01**	NS/NS /**0.05**
**TOTAL PROBLEMS**	50.92 ± 28.05	46.23 ± 29.33	43.00 ± 17.76	NS	54.81 ± 27.24	48.06 ± 31.37	46.48 ± 35.33	NS	NS	36.56 ± 24.13	46.16 ± 26.83	44.92 ± 26.87	NS	36.56 ± 29.08	46.16 ± 23.11	44.92 ± 31.81	NS	NS	**0.05**/NS /NS	**0.05**/NS /NS
**AFFECTIVE PROBLEMS**	70.04 ± 14.98	63.97 ± 16.79	61.50 ± 9.32	NS	68.55 ± 15.55	65.22 ± 17.88	68.10 ± 18.16	NS	NS	59.69 ±11.19	60.88 ± 13.05	56.92 ± 6.23	NS	59.84 ± 16.41	60.88 ± 13.69	56.92 ± 16.41	NS	NS	**0.01**/NS /NS	NS/NS /NS
**ANXIETY PROBLEMS**	64.57 ± 17.95	64.90 ± 16.24	58.63 ± 11.90	NS	65.45 ± 14.70	63.50 ± 16.04	66.57 ± 16.49	NS	NS	60.04 ± 14.15	59.60 ± 15.42	65.83 ± 14.84	NS	60.04 ± 13.66	59.60 ± 13.78	65.83 ± 17.28	NS	NS	NS/ **0.05**/NS	NS/NS /NS

**SOMATIC PROBLEMS**	64.43 ± 15.57	64.10 ± 14.40	54.88 ± 8.16	NS	58.87 ± 12.04	64.25 ± 15.52	59.76 ± 14.18	NS	NS	59.00 ± 12.15	61.60 ± 9.97	59.50 ± 12.35	NS	59.00 ± 4.65	61.60 ± 13.70	59.50 ± 15.85	NS	NS	**0.05**/NS /NS	NS/NS /NS
**ATTENTION DEFICIT-HYPERACTIVITY PROBLEMS**	61.57 ± 13.18	61.35 ± 12.21	61.50 ± 10.39	NS	65.77 ± 12.51	61.59 ± 11.85	64.62 ± 18.12	NS	NS	58.16 ± 12.35	58.80 ± 10.31	65.83 ± 14.69	NS	58.16 ± 15.93	58.80 ± 11.95	65.83 ± 4.46$^{\#}$	**0.01**	NS	NS/**0.05** /NS	NS/NS /NS
**OPPOSITIONAL DEFIANT PROBLEMS**	63.49 ± 15.13	58.55 ± 9.35	59.75 ± 9.39	NS	64.844 ± 13.18	62.81 ± 14.16	66.52 ± 18.04	NS	NS	58.40 ± 9.74	65.64 ± 16.70	65.08 ± 15.45	NS	58.40 ± 14.61	65.64 ± 14.75	65.08 ± 15.07	NS	NS	NS/ NS/ NS	NS/NS /NS
**CONDUCT PROBLEMS**	59.88 ± 13.80	59.39 ± 13.98	54.50 ± 3.33	NS	62.58 ± 16.01	62.31 ± 16.63	61.29 ± 17.03	NS	NS	57.32 ± 10.84	61.68 ± 16.56	59.17 ± 11.77	NS	57.32 ± 7.91	61.68 ± 14.10	59.17 ± 3.77^{+}	**0.05**	NS	NS/ NS/NS	NS/**0.05/ 0.05**
**OBSESSIVE COMPULSIVE PROBLEMS**	64.12 ± 16.21	65.10 ± 16.24	62.63 ± 14.26	NS	61.90 ± 14.40	65.16 ± 16.27	67.29 ± 16.49	NS	NS	63.52 ± 18.53	61.56 ± 13.07	64.75 ± 14.40	NS	63.52 ± 16.41	61.56 ± 16.69	64.75 ± 16.51	NS	NS	NS/**0.05**/ NS	NS/NS /NS
**POST TRAUMATIC STRESS PROBLEMS**	61.24 ± 14.16	61.94 ± 16.70	58.63 ± 8.56	NS	66.00 ± 14.96	64.34 ± 17.14	67.14 ± 19.23	NS	NS	57.36 ± 13.38	62.92 ± 14.81	63.17 ± 14.54	NS	57.36 ± 16.59	62.92 ± 14.50	63.17 ± 17.44	NS	NS	NS/NS/NS	NS/NS/NS
**POSITIVE QUALITIES**	60.18 ± 27.22	65.94 ± 24.04	71.00 ± 21.74	NS	53.32 ± 20.07	56.06 ± 10.17	59.38 ± 27.94	NS	NS	58.20 ± 27.25	63.04 ± 26.92	74.25 ± 18.16	NS	58.20 ± 23.25	63.04 ± 27.74	74.25 ± 29.76	NS	NS	NS/NS/NS	**0.05**/NS/NS

* All results are presented as mean ± SD. Subjects were classified as obese, overweight, or with normal BMI according to IOTF criteria at initial assessment. Tables present comparisons among three groups at both initial and annual assessment. All measured variables were compared by employing repeated-measures ANOVA. Significant main effects were revealed by the LSD posthoc test. Statistical significance was set at ($p < 0.05$, rounded to 0.05 in Table), while strong significance ($p < 0.01$, rounded to 0.01 in Table) is also noted. NS: nonsignificant ($p > 0.05$) difference. +: Indicates significant difference from Overweight group, #: Indicates significant difference from Obese group. p-values between two timepoints refer to obese, overweight, and normal BMI respectively.

Supplemental Table S7. Predictors of psychometric score change in CBCL.

CBCL	Anthropometric Parameters							Metabolic Syndrome					Glucose Metabolism				Pituary Function					Peripheral Hormones					
	Differences of							Differences of					Differences of				Differences of					Differences of					
	Weight	Height	BMI	WC	HC	WHR	WHtR	Glucose	SBP	WC	Tryglycerides	HDL	Glucose	Insulin	HbA1C	HOMA-IR	TSH	PRL	LH	FSH	ACTH	IGF-1	FT4	DHEAS	E2	testosterone	cortisol
**ACTIVITIES**	N/A	N/A	N/A	N/A	N/A	N/A	N/A	N/A	N/A	N/A	N/A	N/A	N/A	N/A	N/A	N/A	N/A	N/A	N/A	N/A	N/A	N/A	N/A	**β=-0.144 (95% CI: -0.201, -0.025)**	N/A	N/A	N/A
**SOCIAL**	N/A	N/A	N/A	N/A	N/A	N/A	N/A	N/A	N/A	N/A	N/A	N/A	N/A	N/A	N/A	N/A	N/A	N/A	N/A	N/A	N/A	N/A	N/A	N/A	N/A	N/A	N/A
**SCHOOL**	N/A	**β=0.155 (95% CI: 0.250, 2.468)**	N/A	N/A	N/A	N/A	**β= -0.142 (95% CI: -108.17, -6.26)**	N/A	N/A	N/A	N/A	N/A	N/A	N/A	N/A	N/A	N/A	N/A	N/A	N/A	N/A	N/A	N/A	N/A	**β=0.133 (95% CI: 0.009, 0.123)**	N/A	N/A
**TOTAL COMPETENCE**	N/A	N/A	N/A	N/A	N/A	N/A	N/A	N/A	N/A	N/A	N/A	N/A	N/A	N/A	N/A	N/A	N/A	N/A	N/A	N/A	N/A	N/A	N/A	N/A	N/A	N/A	N/A
**ANXIOUS-DEPRESSED**	N/A	N/A	N/A	N/A	N/A	N/A	N/A	N/A	N/A	N/A	N/A	N/A	N/A	**β=0.167 (95% CI: 0.119, 0.621)**	N/A	N/A	**β=0.134 (95% CI: 0.163, 1.225)**	N/A	N/A	**β=0.135 (95% CI: -0.802, -0.140)**	N/A	N/A	N/A	N/A	N/A	N/A	N/A
**WITHDRAWN-DEPRESSED**	N/A	N/A	N/A	**β=0.130 (95% CI: 0.015, 0.583)**	N/A	N/A	N/A	N/A	N/A	N/A	N/A	N/A	N/A	N/A	N/A	N/A	N/A	N/A	N/A	**β=0.119 (95% CI: -0.663, -0.075)**	N/A	N/A	N/A	N/A	N/A	N/A	N/A
**SOMATIC COMPLAINTS**	N/A	N/A	N/A	N/A	N/A	N/A	N/A	N/A	N/A	N/A	N/A	N/A	N/A	N/A	N/A	N/A	**β=0.130 (95% CI: 0.076, 0.916)**	N/A	N/A	**β=-0.129 (95% CI: -0.583, -0.057)**	N/A	N/A	N/A	N/A	N/A	N/A	N/A
**SOCIAL PROBLEMS**	N/A	N/A	N/A	N/A	N/A	N/A	N/A	N/A	N/A	N/A	N/A	N/A	N/A	**β=0.156**	N/A	N/A	N/A	N/A	N/A	N/A	N/A	N/A	N/A	N/A	N/A	**β=-0.148**	N/A

CBCL	Anthropometric Parameters							Metabolic Syndrome					Glucose Metabolism				Pituary Function					Peripheral Hormones					
	Differences of							Differences of					Differences of				Differences of					Differences of					
	Weight	Height	BMI	WC	HC	WHR	WHtR	Glucose	SBP	WC	Tryglycerides	HDL	Glucose	Insulin	HbA1C	HOMA-IR	TSH	PRL	LH	FSH	ACTH	IGF-1	FT4	DHEAS	E2	testosterone	cortisol
														(95% CI: 0.083, 0.521)												(95% CI: -0.064, 0.010)	
THOUGHT PROBLEMS	N/A	β=0.165 (95% CI: 0.296, 2.052)	N/A	N/A	N/A	N/A	N/A	N/A	N/A	N/A	β=0.192 (95% CI: 0.010, 0.076)	N/A	N/A	N/A	N/A	N/A	N/A	N/A	β=0.127 (95% CI: 0.018, 0.402)	N/A	N/A	N/A	N/A	N/A	N/A	N/A	β=0.135 (95% CI: 0.057, 0.575)
ATTENTION PROBLEMS	N/A	N/A	N/A	β=0.127 (95% CI: 0.003, 0.423)	N/A	N/A	N/A	β= -0.175 (95% CI: -0.506, -0.048)	N/A	β=0.168 (95% CI: 0.038, 0.516)	N/A	N/A	N/A	N/A	N/A	N/A	N/A	N/A	N/A	β=0.119 (95% CI: 0.061, 0.575)	N/A	N/A	N/A	N/A	β=-0.126 (95% CI: 0.079, 0.005)	N/A	N/A
RULE BREAKING BEHAVIOUR	N/A	N/A	N/A	N/A	N/A	N/A	N/A	N/A	N/A	N/A	N/A	N/A	N/A	N/A	N/A	N/A	N/A	N/A	N/A	N/A	N/A	N/A	N/A	N/A	N/A	N/A	N/A
AGGRESSIVE BEHAVIOUR	N/A	N/A	N/A	N/A	N/A	N/A	N/A	N/A	N/A	N/A	N/A	N/A	N/A	N/A	N/A	N/A	N/A	N/A	N/A	N/A	N/A	N/A	N/A	N/A	N/A	N/A	β=0.155 (95% CI: 0.099, 0.593)
INTERNALIZING PROBLEMS	N/A	N/A	N/A	N/A	N/A	N/A	N/A	N/A	N/A	N/A	N/A	N/A	N/A	N/A	N/A	N/A	β=0.114 (95% CI: 0.286, 2.430)	N/A	N/A	β=0.180 (95% CI: 0.343, 1.749)	N/A	N/A	N/A	N/A	N/A	N/A	N/A
EXTERNALIZING PROBLEMS	N/A	N/A	N/A	N/A	N/A	N/A	N/A	N/A	N/A	N/A	N/A	N/A	N/A	N/A	N/A	N/A	N/A	N/A	N/A	β=0.129 (95% CI: 0.142 1.812)	N/A	N/A	N/A	N/A	N/A	N/A	β=0.131 (95% CI: 0.081, 0.963)
TOTAL PROBLEMS	N/A	N/A	N/A	N/A	N/A	N/A	N/A	N/A	N/A	N/A	N/A	N/A	N/A	N/A	N/A	N/A	β=0.141	N/A	N/A	β=0.168	N/A	N/A	N/A	N/A	N/A	β=-0.118	β=0.123

CBCL	Anthropometric Parameters							Metabolic Syndrome					Glucose Metabolism				Pituary Function					Peripheral Hormones					
	Differences of							Differences of					Differences of				Differences of					Differences of					
	Weight	Height	BMI	WC	HC	WHR	WHtR	Glucose	SBP	WC	Tryglycerides	HDL	Glucose	Insulin	HbA1C	HOMA-IR	TSH	PRL	LH	FSH	ACTH	IGF-1	FT4	DHEAS	E2	testosterone	cortisol
																	(95% CI: 0.232, 4.292)			(95% CI: 0.405, 3.117)						(95% CI: -0.096, -0.002)	(95% CI: 0.051, 0.979)
AFFECTIVE PROBLEMS	N/A	N/A	N/A	N/A	N/A	N/A	β=0.155 (95% CI: 12.11, 15.87)	N/A	N/A	β=0.148 (95% CI: 0.075, 0.773)	N/A	N/A	N/A	N/A	β=0.131 (95% CI: 0.76, 0.92)	N/A	β=0.184 (95% CI: 0.212, 1.130)	N/A	N/A	β=0.188 (95% CI: 0.073, 0.653)	N/A	N/A	N/A	N/A	N/A	β=-0.135 (95% CI: -0.070, -0.008)	β=0.152 (95% CI: 0.133, 0.785)
ANXIETY PROBLEMS	N/A	N/A	N/A	N/A	N/A	N/A	N/A	N/A	N/A	N/A	N/A	N/A	N/A	N/A	N/A	N/A	N/A	N/A	N/A	N/A	N/A	N/A	N/A	N/A	N/A	N/A	N/A
SOMATIC PROBLEMS	N/A	N/A	N/A	N/A	N/A	N/A	N/A	N/A	N/A	N/A	N/A	N/A	N/A	N/A	N/A	N/A	N/A	N/A	N/A	N/A	N/A	N/A	N/A	N/A	N/A	N/A	N/A
ATTENTION DEFICIT-HYPERACTIVITY PROBLEMS	N/A	N/A	N/A	β=0.128 (95% CI: 0.008, 0.506)	N/A	N/A	N/A	N/A	N/A	N/A	N/A	N/A	N/A	β=0.158 (95% CI: 0.081, 0.477)	N/A	N/A	β=0.115 (95% CI: 0.079, 1.181)	N/A	N/A	N/A	N/A	N/A	N/A	N/A	N/A	N/A	β=0.125 (95% CI: 0.033, 0.539)
OPPOSITIONAL DEFIANT PROBLEMS	N/A	N/A	N/A	N/A	N/A	N/A	N/A	N/A	N/A	N/A	N/A	N/A	β=0.132 (95% CI: 0.03, 0.41)	N/A	N/A	N/A	β=0.174 (95% CI: 0.117, 0.841)	N/A	N/A	N/A	β= -0.121 (95% CI: -0.122, -0.004)	N/A	N/A	N/A	N/A	N/A	N/A
CONDUCT PROBLEMS	N/A	N/A	N/A	N/A	N/A	N/A	N/A	N/A	N/A	N/A	N/A	N/A	N/A	N/A	N/A	N/A	N/A	N/A	N/A	N/A	N/A	N/A	N/A	N/A	N/A	β=-0.160 (95% CI: -0.063, -0.011)	β=0.154 (95% CI: 0.105, 0.623)
SLUGGISH COGNITIVE PROBLEMS	N/A	N/A	N/A	N/A	N/A	N/A	N/A	N/A	N/A	N/A	N/A	N/A	N/A	N/A	N/A	N/A	N/A	N/A	N/A	N/A	N/A	N/A	N/A	N/A	N/A	N/A	N/A

CBCL	Anthropometric Parameters							Metabolic Syndrome					Glucose Metabolism				Pituary Function					Peripheral Hormones					
	Differences of							Differences of					Differences of				Differences of					Differences of					
	Weight	Height	BMI	WC	HC	WHR	WHtR	Glucose	SBP	WC	Tryglycerides	HDL	Glucose	Insulin	HbA1C	HOMA-IR	TSH	PRL	LH	FSH	ACTH	IGF-1	FT4	DHEAS	E2	testosterone	cortisol
**OBSESSIVE COMPULSIVE PROBLEMS**	N/A	N/A	N/A	N/A	N/A	N/A	N/A	N/A	N/A	N/A	N/A	N/A	N/A	N/A	N/A	N/A	**N/A**	N/A	N/A	N/A	N/A	N/A	N/A	N/A	N/A	N/A	N/A
**POST-TRAUMATIC STRESS PROBLEMS**	N/A	N/A	N/A	**β=0.135 (95% CI: 0.025, 0.569)**	N/A	N/A	N/A	N/A	N/A	N/A	N/A	N/A	N/A	N/A	N/A	N/A	**β=0.166 (95% CI: 0.885, 3.527)**	N/A	N/A	**β=0.130 (95% CI: 0.695, 2.655)**	N/A	N/A	N/A	N/A	N/A	N/A	N/A

- β= beta co-efficient, CI: confidence interval

Supplemental Table S8. Predictors of psychometric score change after the lifestyle intervention in YSR.

YSR	Anthropometric Parameters							Metabolic Syndrome					Glucose Metabolism				Pituary Function					Peripheral Hormones					
	Differences of							Differences of					Differences of				Differences of					Differences of					
	Weight	Height	BMI	WC	HC	WHR	WHtR	Glucose	SBP	WC	Tryglycerides	HDL	Glucose	Insulin	HbA1C	HOMA-IR	TSH	PRL	LH	FSH	ACTH	IGF-1	FT4	DHEAS	E2	testosterone	cortisol
**ACTIVITIES**	N/A	β= 0.130 (95% CI: 0.033, 0.293)	N/A	N/A	N/A	N/A	N/A	N/A	N/A	N/A	N/A	N/A	N/A	N/A	N/A	N/A	N/A	N/A	N/A	N/A	N/A	N/A	N/A	N/A	N/A	N/A	N/A
**SOCIAL**	N/A	N/A	N/A	N/A	N/A	N/A	N/A	N/A	N/A	N/A	N/A	N/A	N/A	N/A	N/A	N/A	N/A	N/A	N/A	N/A	N/A	N/A	N/A	N/A	N/A	N/A	N/A
**TOTAL COMPETENCE**	N/A	N/A	N/A	N/A	N/A	N/A	N/A	N/A	N/A	N/A	N/A	N/A	N/A	N/A	N/A	N/A	N/A	N/A	N/A	N/A	N/A	N/A	N/A	N/A	N/A	N/A	N/A
**ANXIOUS-DEPRESSED**	N/A	N/A	N/A	N/A	N/A	N/A	N/A	N/A	N/A	N/A	N/A	N/A	N/A	N/A	β=0.207 (95% CI: 0.10, 0.30)	N/A	N/A	N/A	N/A	N/A	N/A	N/A	N/A	N/A	N/A	N/A	N/A
**WITHDRAWN-DEPRESSED**	N/A	N/A	N/A	β=-0.239 (95% CI: -0.939, -0.037)	N/A	N/A	N/A	N/A	N/A	N/A	β=0.333 (95% CI: 0.080, 0.169)	N/A	N/A	N/A	N/A	N/A	N/A	N/A	N/A	N/A	N/A	β=-0.233 (95% CI: -0.039, -0.003)	N/A	N/A	N/A	N/A	N/A
**SOMATIC COMPLAINTS**	N/A	N/A	N/A	N/A	N/A	N/A	N/A	N/A	N/A	N/A	N/A	N/A	N/A	N/A	N/A	N/A	N/A	N/A	N/A	N/A	N/A	N/A	N/A	N/A	N/A	N/A	N/A
**SOCIAL PROBLEMS**	N/A	N/A	N/A	N/A	N/A	N/A	N/A	β=-0.273 (95% CI: -	N/A	N/A	β=0.365 (95% CI: 0.020, 0.106)	β=0.309 (95 %	N/A	N/A	β=0.268 (95% CI:	N/A	β=0.202 (95% CI:	N/A	N/A	N/A	N/A	N/A	N/A	N/A	N/A	N/A	N/A

YSR	Anthropometric Parameters							Metabolic Syndrome					Glucose Metabolism				Pituary Function					Peripheral Hormones					
	Differences of							Differences of					Differences of				Differences of					Differences of					
	Weight	Height	BMI	WC	HC	WHR	WHtR	Glucose	SBP	WC	Tryglycerides	HDL	Glucose	Insulin	HbA1C	HOMA-IR	TSH	PRL	LH	FSH	ACTH	IGF-1	FT4	DHEAS	E2	testosterone	cortisol
								0.988, -0.058)				CI: 0.146, 1.282)			0.53, 0.95)		0.12, 6.11)										
**THOUGHT PROBLEMS**	N/A	N/A	N/A	N/A	N/A	N/A	N/A	N/A	N/A	N/A	β=0.408 (95% CI: 0.025, 0.095)	β=0.366 (95% CI: 0.234, 1.186)	β=-0.223 (95% CI: -0.609, -0.041)	N/A	N/A	N/A	N/A	N/A	N/A	N/A	N/A	N/A	N/A	N/A	N/A	N/A	N/A
**ATTENTION PROBLEMS**	N/A	N/A	N/A	N/A	N/A	N/A	N/A	β=-0.272 (95% CI: -0.805, -0.025)	N/A	N/A	β=0.298 (95% CI: 0.006, 0.076)	N/A	N/A	N/A	β=0.255 (95% CI: 0.507, 0.990)	N/A	N/A	N/A	N/A	N/A	N/A	N/A	N/A	β=0.207 (95% CI: 0.004, 0.106)	N/A	N/A	N/A
**RULE BREAKING BEHAVIOUR**	N/A	N/A	N/A	N/A	N/A	N/A	N/A	N/A	N/A	N/A	N/A	β=0.282 (95% CI: 0.122, 0.768)	N/A	N/A	N/A	N/A	N/A	β= -0.254 (95% CI: -0.82, -0.07)	β=0.404 (95% CI: 0.23, 0.73)	N/A	N/A	N/A	N/A	N/A	N/A	N/A	N/A
**AGGRESSIVE BEHAVIOUR**	N/A	N/A	N/A	N/A	N/A	N/A	N/A	N/A	N/A	N/A	N/A	β=0.335 (95% CI: 0.154,	N/A	N/A	N/A	N/A	N/A	β= -0.303 (95% CI: -	N/A	N/A	N/A	N/A	N/A	N/A	N/A	N/A	N/A

YSR	Anthropometric Parameters							Metabolic Syndrome					Glucose Metabolism				Pituary Function					Peripheral Hormones					
	Differences of							Differences of					Differences of				Differences of					Differences of					
	Weight	Height	BMI	WC	HC	WHR	WHtR	Glucose	SBP	WC	Tryglycerides	HDL	Glucose	Insulin	HbA1C	HOMA-IR	TSH	PRL	LH	FSH	ACTH	IGF-1	FT4	DHEAS	E2	testosterone	cortisol
												1.158)						1.29, -0.29)									
**INTERNALIZING PROBLEMS**	N/A	N/A	N/A	N/A	N/A	N/A	N/A	N/A	N/A	N/A	N/A	N/A	N/A	N/A	**β=0.233 (95% CI: 0.070, 0.780)**	N/A	N/A	N/A	N/A	N/A	N/A	**β=-0.213 (95% CI: -0.061, -0.003)**	N/A	N/A	N/A	N/A	N/A
**EXTERNALIZING PROBLEMS**	N/A	N/A	N/A	N/A	N/A	N/A	N/A	N/A	N/A	N/A	N/A	N/A	N/A	N/A	N/A	N/A	N/A	**β= -0.393 (95% CI: -2.48, -0.74)**	**β=0.256 (95% CI: 0.12, 0.128)**	N/A	N/A	N/A	N/A	N/A	N/A	N/A	N/A
**TOTAL PROBLEMS**	N/A	N/A	N/A	N/A	N/A	N/A	N/A	N/A	N/A	N/A	N/A	N/A	N/A	N/A	**β=0.288 (95% CI: 0.420, 0.978)**	N/A	N/A	N/A	N/A	N/A	N/A	N/A	N/A	N/A	N/A	N/A	N/A
**AFFECTIVE PROBLEMS**	N/A	N/A	N/A	N/A	N/A	N/A	N/A	N/A	N/A	N/A	N/A	N/A	N/A	N/A	**β=0.268 (95% CI: 0.079, 0.548)**	N/A	N/A	**β= -0.207 (95% CI: -**	N/A	N/A	N/A	**β= -0.254 (95% CI: -0.04**	N/A	N/A	N/A	N/A	N/A

YSR	Anthropometric Parameters							Metabolic Syndrome					Glucose Metabolism				Pituary Function					Peripheral Hormones					
	Differences of							Differences of					Differences of				Differences of					Differences of					
	Weight	Height	BMI	WC	HC	WHR	WHtR	Glucose	SBP	WC	Tryglycerides	HDL	Glucose	Insulin	HbA1C	HOMA-IR	TSH	PRL	LH	FSH	ACTH	IGF-1	FT4	DHEAS	E2	testosterone	cortisol
																		1.13, - 0.03)				3, - 0.006)					
ANXIETY PROBLEMS	N/A	N/A	N/A	N/A	N/A	N/A	N/A	N/A	N/A	N/A	N/A	N/A	N/A	N/A	N/A	N/A	N/A	N/A	N/A	N/A	N/A	N/A	N/A	N/A	N/A	N/A	N/A
SOMATIC PROBLEMS	N/A	N/A	N/A	N/A	N/A	N/A	N/A	N/A	N/A	N/A	N/A	N/A	N/A	N/A	N/A	N/A	N/A	N/A	N/A	N/A	N/A	N/A	N/A	N/A	N/A	N/A	N/A
ATTENTION DEFICIT-HYPERACTIVITY PROBLEMS	N/A	N/A	N/A	N/A	N/A	N/A	N/A	β=-0.307 (95% CI: -0.816, -0.100)	N/A	N/A	β=0.421 (95% CI: 0.024, 0.090)	N/A	N/A	N/A	β=0.223 (95% CI: 0.800, 0.987)	N/A	N/A	N/A	N/A	N/A	N/A	N/A	N/A	N/A	N/A	N/A	N/A
OPPOSITIONAL DEFIANT PROBLEMS	N/A	N/A	N/A	N/A	N/A	N/A	N/A	N/A	N/A	N/A	N/A	N/A	N/A	N/A	N/A	N/A	N/A	β= -0.236 (95% CI: -1.13,-0.03)	N/A	N/A	β=0.215 (95% CI: 0.03, 0.42)	N/A	N/A	N/A	N/A	N/A	N/A
CONDUCT PROBLEMS	N/A	N/A	N/A	N/A	N/A	N/A	N/A	β=-0.310 (95% CI: -0.819, 0.095)	N/A	N/A	N/A	β=0.303 (95% CI: 0.101, 0.975)	β=-0.226 (95% CI: -0.617, -0.045	N/A	N/A	N/A	N/A	N/A	N/A	β=0.229 (95% CI: 0.16, 1.92)	N/A	N/A	N/A	N/A	N/A	N/A	N/A
OBSESSIVE COMPULSIVE PROBLEMS	N/A	β=-0.257	N/A	N/A	N/A	N/A	N/A	N/A	N/A	N/A	N/A	N/A	N/A	N/A	N/A	N/A	N/A	N/A	N/A	N/A	N/A	N/A	N/A	N/A	N/A	N/A	N/A

YSR	Anthropometric Parameters							Metabolic Syndrome					Glucose Metabolism				Pituary Function					Peripheral Hormones					
	Differences of							Differences of					Differences of				Differences of					Differences of					
	Weight	Height	BMI	WC	HC	WHR	WHtR	Glucose	SBP	WC	Tryglycerides	HDL	Glucose	Insulin	HbA1C	HOMA-IR	TSH	PRL	LH	FSH	ACTH	IGF-1	FT4	DHEAS	E2	testosterone	cortisol
		**(95% CI: -2.153, -1.650)**																									
**POST TRAUMATIC STRESS PROBLEMS**	N/A	N/A	N/A	N/A	N/A	N/A	N/A	N/A	N/A	N/A	**β=0.323 (95% CI: 0.056, 0.769)**	N/A	N/A	N/A	N/A	N/A	N/A	N/A	N/A	N/A	N/A	**β= -0.221 (95% CI: -0.034, -0.002)**	N/A	N/A	N/A	N/A	N/A
**POSITIVE QUALITIES**	N/A	N/A	N/A	N/A	N/A	N/A	N/A	N/A	N/A	N/A	N/A	N/A	N/A	N/A	N/A	N/A	N/A	N/A	N/A	N/A	N/A	N/A	N/A	N/A	N/A	N/A	N/A

- β= beta co-efficient, CI: confidence interval
